# A Photo-Smiles Rearrangement: Mechanistic Investigation of the Formation of Blatter Radical Helicenes

**DOI:** 10.1021/acs.joc.4c02893

**Published:** 2025-02-05

**Authors:** Hemant K. Singh, Sławomir Kaźmierski, Piotr Kaszyński

**Affiliations:** § Centre of Molecular and Macromolecular Studies, Polish Academy of Sciences, 90-363 Łódź, Poland; ‡ Faculty of Chemistry, University of Łódź, 91-403 Łódź, Poland; # Department of Chemistry, Middle Tennessee State University, Murfreesboro, Tennessee 37130, United States

## Abstract

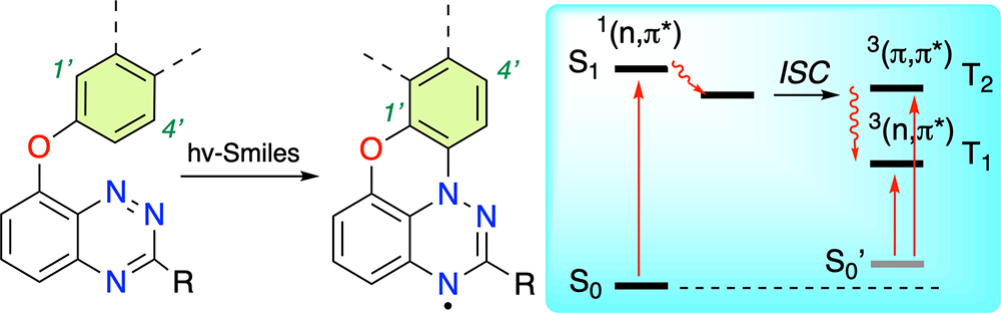

Photocyclization of 8-aryloxy-3-phenylbenzo[*e*][1,2,4]triazines leads to helicene radicals. Structural analysis of radicals *leuco* forms by two-dimensional correlation nuclear magnetic resonance methods demonstrated that the photocyclization involves a Smiles rearrangement and exclusive formation of a single rearranged product for all substrates. Density functional theory investigations indicate that the mechanism requires the T_1_ state with (n, π*) character localized on the benzo[*e*][1,2,4]triazine (BT) fragment and at least one occupied π molecular orbital (MO) localized on the aryloxy fragment with an energy that is higher than that of the n MO. This electronic structure is favorable for aryl-to-BT single-electron transfer and formation of a zwitterion, which undergoes an intramolecular polar cyclization followed by ring opening of the resulting spirooxazole. The proposed mechanism represents a new variation of photo-Smiles rearrangement and appears to be general for the photochemical formation of planar Blatter radicals.

## Introduction

**Figure 1:**
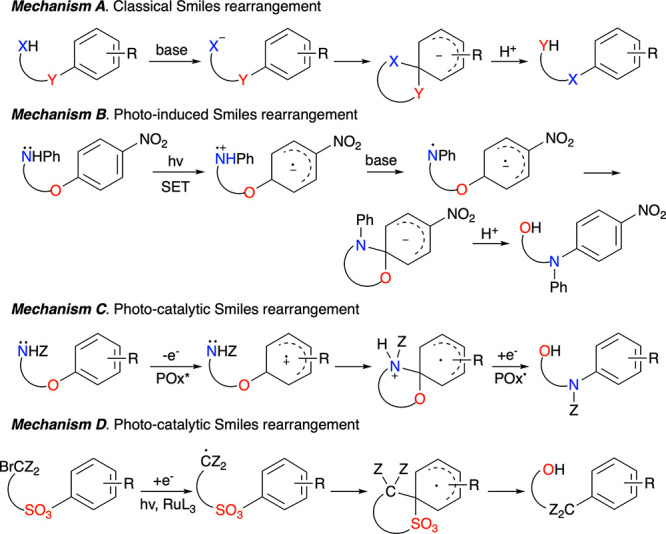
Four general mechanisms of Smiles rearrangement. POx is a photooxidant. For details and references, see the text.

Smiles rearrangement^1−3^ and its variations^4−8^ are among the most common transformations in the chemistry of aromatic compounds and highly useful in organic synthesis.^9,10^ The general mechanism involves intramolecular attack on the *ipso* carbon atom of the arene, formation of a spiro species (either an intermediate or a transition state)^11,12^ followed by ring opening and formation of the rearranged product (Figure 1). The classical rearrangement typically is a polar, thermally activated process (mechanism A in Figure 1)^3^ but can also proceed via a photoinduced radical mechanism.^13−15^

A mechanism involving transient radicals was postulated early on for Smiles rearrangement in compounds with a general structure of RNH(CH_2_)_*n*_OC_6_H_4_NO_2_-4 (mechanism B in Figure 1). Spectroscopic and kinetic investigation demonstrated^16,17^ that the rearrangement consists of the initial photoinduced intramolecular charge transfer (CT) and formation of an open-shell zwitterion (a radical anion, radical cation pair), followed by deprotonation of the amminium nitrogen and intramolecular radical–radical anion recombination. The resulting spirooxazole opens, leading to the rearranged product.^18−20^ More recently, a new Smiles rearrangement protocol was developed (mechanism C in Figure 1), in which the electrophilic aryloxy radical cation is generated by a photoinduced intermolecular single-electron transfer (SET)^21^ to an electron acceptor [photooxidant (POx), e.g., acridinium, pyrylium, or 9,10-dicyanoanthracene].^22^ The subsequent intramolecular nucleophilic addition leads to the spirooxazole, which opens to yield the rearrangement product.

Another photoinduced radical Smiles rearrangement involves a Ru photocatalyst. In this case, the catalyst effects one-electron reduction of an alkyl bromide, leading to the formation of the corresponding alkyl radical, which attacks the *ipso* atom and displaces the sulfonyloxy group (mechanism D in Figure 1).^13^

**Figure 2:**
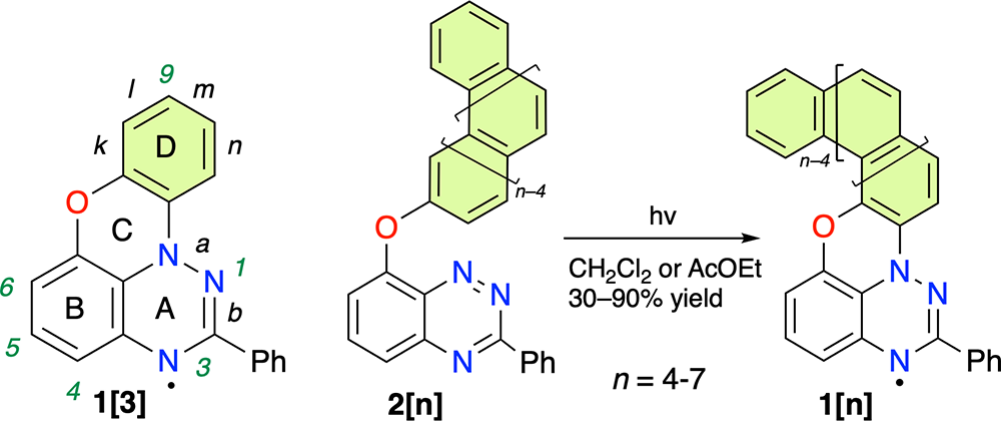
Structures of prototypical planar Blatter radical **1[3]** with a partial numbering system and photocyclization of **2[*n*]** to radicals **1[*n*]**.^25^

Several years ago, we demonstrated that the prototypical planar Blatter radical, triazino[5,6,1-*kl*]phenoxazinyl **1[3]** (Figure 2), and its derivatives can be obtained by irradiation of dilute solutions of the appropriate 8-aryloxybenzo[*e*][1,2,4]triazines **2[*n*]** in yields that depend on the structure of the aryloxy fragment and solvent.^23,24^ Although the initial analysis suggested a simple cyclization mechanism, most recent crystallographic studies in series **1[*n*]** indicate, however, that the obtained radicals result from a Smiles-type rearrangement and formal migration of the oxygen atom to the adjacent position in the aryl fragment (Figure 2).^25^ Unlike those of other photoinduced Smiles rearrangements, the reaction medium does not contain a base (as in mechanism B in Figure 1) or a photoactive redox additive, as in mechanisms C and D. This suggests a different mechanism for the observed rearrangements.

Herein, we report a complete mechanistic analysis of the photocyclization of 8-aryloxybenzo[*e*][1,2,4]triazines **2[*n*]** and the formation of **1[*n*]** (Figure 2) in a surprising photo-Smiles rearrangement process. The regioselectivity of this process is determined by two-dimensional (2D) nuclear magnetic resonance (NMR) methods. Our results provide the basis for understanding the photocyclization of other benzo[*e*][1,2,4]triazine derivatives, leading to a broad family of planar polycyclic stable radicals. The mechanistic and experimental NMR analyses are supported with density functional theory (DFT) computational results.

## Results

**Scheme 1:**
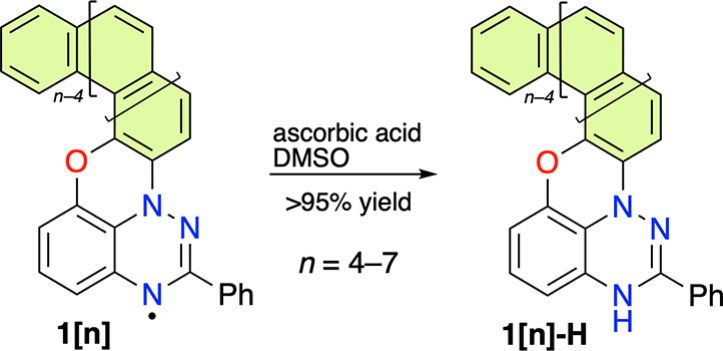
Preparation of *leuco* Derivatives **1[*n*]-H**

We initially aimed to establish the structure of all radicals in series **1[*n*]** and to determine the structural uniformity of the bulk samples using ^1^H–^1^H NMR correlation analysis of *leuco* derivatives **1[*n*]-H**. The former analysis complements the X-ray diffraction structures of **1[5]** and **1[7]**,^25^ while the latter issue is important for determining the degree of regioselectivity of the photocyclization process. The requisite *leuco***1[*n*]-H** forms were obtained by reduction of radicals **1[*n*]** with ascorbic acid in DMSO-*d*_6_ containing a drop of CD_2_Cl_2_ and D_2_O (Scheme 1).

### NMR Structural Analysis of **1[*n*]-H**

**Figure 3:**
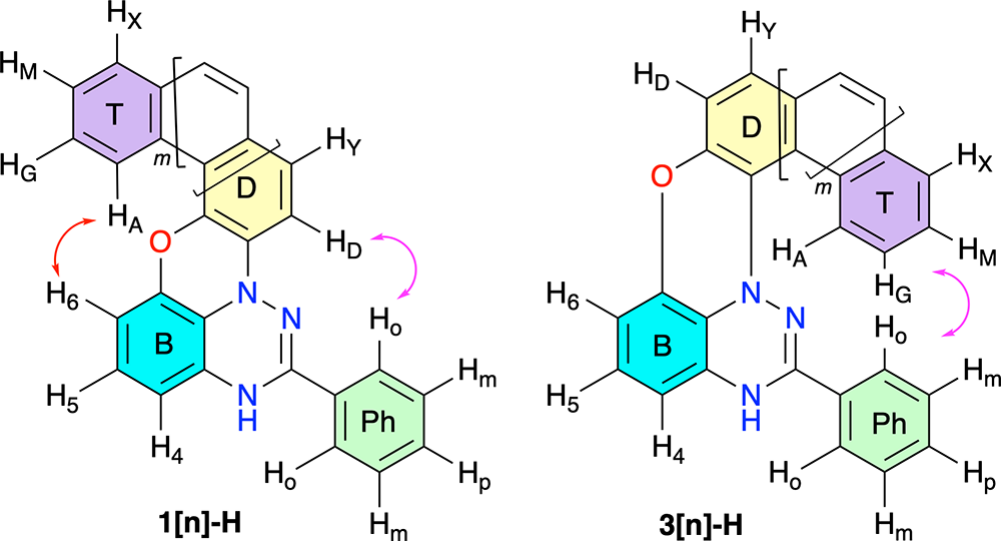
Four distinct spin systems in *leuco* derivatives **1[*n*]-H** and **3[*n*]-H**. The red arrows indicate significant through-space H···H interactions.

^1^H NMR spectra of freshly prepared samples of **1[*n*]-H** revealed single species with generally well-resolved multiplets in the aromatic region. In each compound, there are four distinct spin systems, which were identified by H–H correlation spectroscopy (COSY and TOCSY^26^) on the basis of their characteristic number of interacting hydrogen nuclei and coupling patterns (Figure 3 and the Supporting Information). The subsequent analysis using the ROSEY^27^ method in combination with DFT molecular modeling identified key through-space interactions.

The results demonstrate communication of the four-spin system of terminal benzene ring T (H_A_H_G_H_M_H_X_) with the high-field three-spin system of ring B (H_4_H_5_H_6_) in all derivatives, which is consistent with structure **1[*n*]-H**. In the two lower homologues, ROESY detected weak through-space interactions only between H_A_ and H_6_ hydrogen atoms of rings T and B, respectively [DFT H···H distances of 3.85 Å in **1[4]-H** and 2.87 Å in **1[5]-H** (see the Supporting Information)], while in **1[6]-H**, H_6_ interacts with H_A_ and H_G_ (3.68 and 3.77 Å, respectively). In **1[7]-H**, H_6_ interacts with the entire spin system of ring T. In addition, H_X_ interacts with H_D_ of ring D (4.83 Å). The observed interactions in higher homologues result from progressive overlap of terminal rings T and B.

Further support for this assignment is provided by through-space interactions of the two-spin system of ring D with the five-spin system of the phenyl ring [Ph (Figure 3)] observed in homologues **1[4]-H** and **1[7]-H** (DFT H_o_···H_4_ distances 3.17 and 3.14 Å, respectively). The interactions described above are absent in isomeric *leuco* derivatives **3[*n*]-H**, in which only spin systems of terminal ring T and phenyl ring Ph could interact through space.

The structural assignment to **1[*n*]-H** is consistent with the observed trends in chemical shifts δ caused by gradually increasing the proximity and overlap of terminal rings B and T. Thus, while the δ of H_6_ and H_5_ is in the expected range of ∼6.7 ppm in **1[4]-H** and **1[5]-H**, in higher homologues the protons are increasingly shielded by the anisotropic current of ring T. The most complete overlap and most effective shielding are observed for H_6_ in **1[7]-H** with δ = 4.51 (Table 1). It also needs to be mentioned that H_A_ in nearly planar **1[5]-H** is strongly deshielded (δ = 9.42 ppm) by close contacts (H-bonding) with the O(7) atom (DFT H···O distance of 2.04 Å).

**Table 1:** Chemical Shifts of Characteristic Protons of *leuco***1[*n*]-H**^a^

*leuco*	H_4_ (d)	H_5_ (t)	H_6_ (d)	H_A_ (d)	H_G_ (t)	H_M_ (t)	H_X_ (d)	H_D_ (d)
**1[4]-H**	6.39	6.76	6.53	7.89	7.46	7.35	7.77	7.59
**1[5]-H**	6.37	6.71	6.75	9.42	7.61	7.61	7.88	7.64
**1[6]-H**	6.36	6.67	5.88	8.18	7.49	7.59	8.01	7.67
**1[7]-H**	6.11	6.22	4.51	8.10	7.27	7.36	7.99	8.07

a Recorded in DMSO-*d*_6_. Multiplicities of the signal are given in parentheses.

The observed trends in chemical shifts are well reproduced by DFT calculations at the B3LYP/6-311G(2d,p)//B3LYP/6-31G(2d,p) level of theory in DMSO dielectric medium with an overall correlation parameter *r*^2^ of 0.96.^28^ In particular, the characteristically outstanding shifts for H_A_ in **1[5]-H** (δ_exp_ = 9.42 ppm, and δ_DFT_ = 9.48 ppm) and H_6_ in **1[6]-H** (δ_exp_ = 5.88 ppm, and δ_DFT_ = 5.55 ppm) and **1[7]-H** (δ_exp_ = 4.51 ppm, and δ_DFT_ = 4.60 ppm) are reasonably well predicted.^28^

The NMR analyses described above demonstrate that the photocyclization process is fully regioselective for the formation of rearranged product **1[*n*]**, while other, nonrearranged products (such as **3[*n*]**) are completely absent. These results indicate that the structural assignments of some of the previously reported photocyclization products need to be revisited.^23,24^

### Mechanistic Analysis

**Figure 4:**
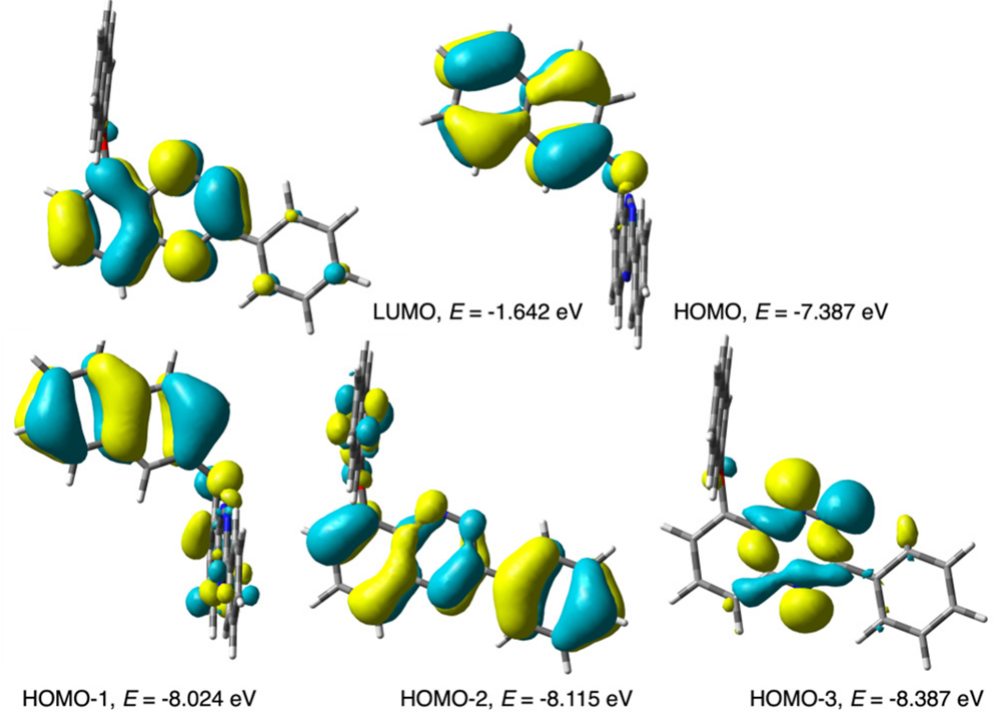
Selected CAM-B3LYP/6-311G(d,p)-derived MO contours and energies relevant to low-energy excitations of **2[4]** at the GS equilibrium geometry (MO isovalue of 0.03). For the sake of clarity, two orientations of **2[4]** are used.

Photocyclization of **2[*n*]** and formation of **1[*n*]** were investigated with DFT methods using **2[4]** as an example. Calculations at the TD-CAM-B3LYP/6-311G(d,p) level of theory in the EtOAc dielectric medium indicate that the lowest-energy excitation, S_0_ → S_1_, in **2[4]** at the GS conformational minimum takes place at 441 nm with an oscillator strength *f* of ≈0.004 and has ^1^(n, π*) character. It involves mainly (∼86%) the transition from HOMO–3, encompassing all three nonbonding electron pairs, to the LUMO, both localized on the benzo[*e*][1,2,4]triazine (Figure 4). Relaxation of the Franck–Condon geometry to the equilibrium geometry of the S_1_ state is exothermic by a Δ*E*_SCF_ of 6.3 kcal mol^–1^ (0.26 eV). The relaxed S_1_ state still has ^1^(n, π*) character in which the n molecular orbital (MO) density partially extends from the benzo[*e*][1,2,4]triazine to the aryloxy substituent.

**Figure 5:**
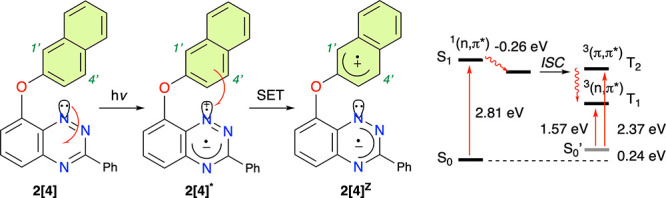
Photoexcitation of **2[4]** to the ^1^(n, π*) state (**2[4]**) and intramolecular electron transfer leading to a charge-separated zwitterionic species (**2[4]**^**Z**^) (left). Partial Jablonski diagram for the generation of the ^3^(n, π*) state (**2[4]**^**3**^) (right).

The ^1^(n, π*) state may undergo ISC to a triplet state in a process allowed by the El-Sayed rules^29^ first to the ^3^(π, π*) state, which through internal conversion (IC) relaxes to the T_1_^3^(n, π*) state (Figure 5). For reference, in cinnoline, a close analogue of benzo[*e*][1,2,4]triazine, the S_1_ state decays via ISC with time constants of <232 ps.^30^ The energy of the resulting T_1_ state of **2[4]** is lower by a Δ*E*_SCF_ of 14.6 kcal mol^–1^ (0.54 eV) than that of the S_1_ state (Figure 5). TD-DFT calculations of the forbidden S_0_ → T_1_ excitation at the triplet geometry demonstrate that the T_1_ state also has (n, π*) character and is localized on the benzo[*e*][1,2,4]triazine fragment. Thus, in both states, ^1^(n, π*) and ^3^(n, π*), there is a formal hole on the n orbital and a negative charge is delocalized in the heterocycle (Figure 5).

**Figure 6:**
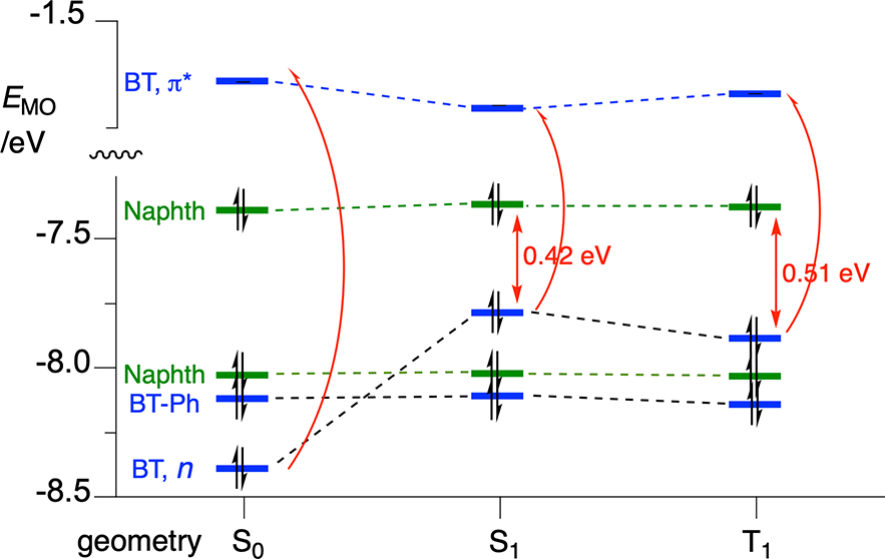
Energy of MOs shown in Figure 4 for three geometries of **2[4]**: in the ground state (S_0_) and relaxed S_1_ and T_1_ states. Colors indicate localization of the MO: blue for the heterocycle (BT) and heterocycle with C(3)-Ph (BT-Ph) and green for naphthalene (Naphth). The red arrow indicates the lowest-energy excitation from the closed-shell ground state.

Analysis of MO energies of the S_0_ state indicates that there are two π orbitals localized on the naphthalene ring, HOMO and HOMO–1, with energies higher than that of the n orbital (Figure 6). In the geometry of the relaxed S_1_ and T_1_ states, the n MO becomes HOMO–1, still significantly below the π HOMO (Figure 6), which provides the thermodynamic driving force for SET and the formation of zwitterion **2[4]**^**Z**^. Thus, in the first approximation, in the S_1_ or T_1_ state, an electron tunnels from the naphthyloxy fragment to fill the formally half-occupied n orbital giving rise to charge-separated zwitterionic species **2[4]**^**Z**^ with a radical anion delocalized in the heterocycle and a radical cation in the aryloxy fragment. This opens up the possibility for subsequent nucleophilic attack of the N(1) atom on the naphthyloxy fragment.

**Figure 7:**
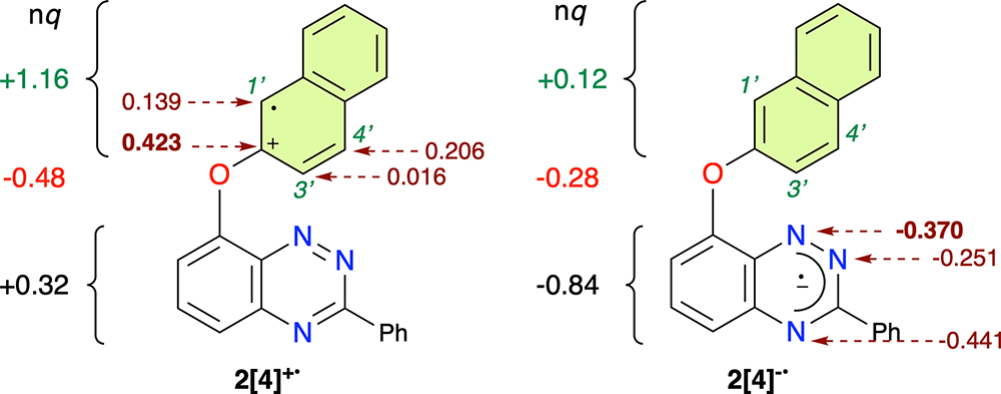
Natural charge (*nq*) density (NBO population analysis) on selected atoms and fragments in radical ions at the S_0_ equilibrium geometry of **2[4]**. UCAM-B3LYP/6-311+G(d,p)//CAM-B3LYP/6-311G(d,p) method in EtOAc dielectric medium. For C(1′), C(3′), and C(4′), the sum of C and H charges is shown.

**Figure 8:**
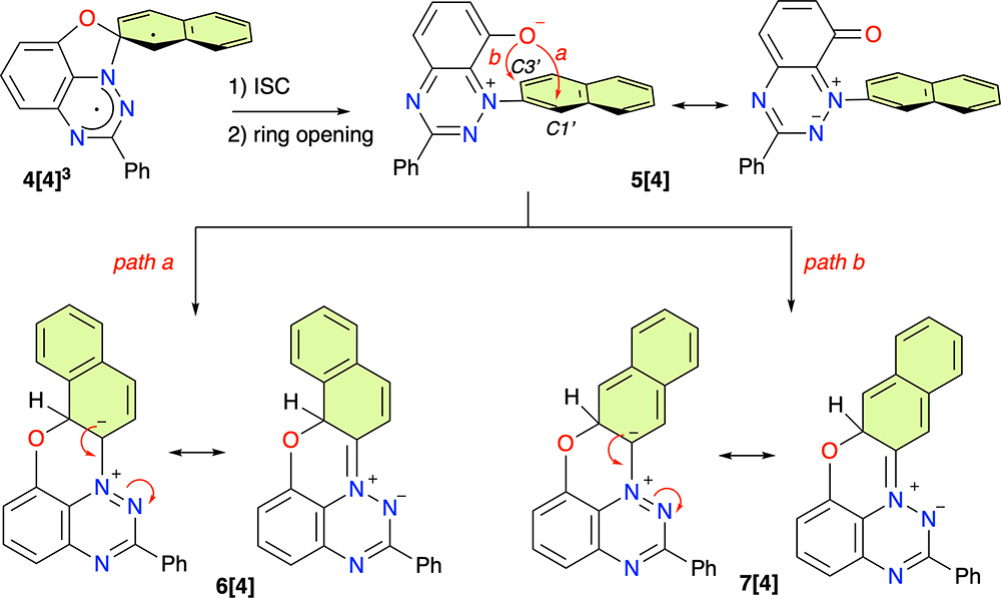
Two paths for the rearrangement of **4[4]**^**3**^ leading to two isomeric intermediates, **6[4]** and **7[4]**.

To assess the regioselectivity of the intramolecular nucleophilic attack, the charge distribution in two radical ions of **2[4]** in its GS equilibrium geometry was analyzed with NBO population analysis. The results show that in radical cation **2[4]**^**+•**^ nearly 90% of the hole is localized on the naphthalene ring with the highest density at the *ipso* carbon (Figure 7). The resonance structure shown in Figure 7 is consistent with the calculated distribution of the charge and spin densities in the naphthalene ring. In the analogous radical anion, the entire additional electron is localized on the heterocycle with the highest natural negative charge on the N(4) (*nq* = −0.414) and N(1) (*nq* = −0.370) atoms. In this case, analysis shows that the spin is delocalized in the π system, while the negative charge is in the σ electron manifold. Thus, on the basis of the charge distribution, the preferred attack takes place on the *ipso* position with the formation of diradical spirooxazole intermediate **4[4]** (Figure 8).

Geometry optimizations located triplet spirooxazole diradical **4[4]**^**3**^ on the potential energy surface, with the stability being greater by a Δ*H* of 1.58 kcal mol^–1^ than that of **2[4]**^**3**^. Attempts to find the analogous spiro structure as a closed- or open-shell singlet led to the oxazole ring opening by rupture of the C(2′)–O bond and formation of zwitterion **5[4]** (Figure 8). A close analogue of **5[4]** was just isolated and characterized by XRD as a side product in photocyclization of **2[9]**, which provide an additional support for the proposed mechanism. DFT calculations demonstrate that the process is exothermic by a Δ*H* of 28.9 kcal mol^–1^, which is largely due to the recovery of one Clar’s sextet.

NBO analysis of zwitterion **5[4]** revealed an increased negative natural charge on the oxygen atom (*nq* = −0.67) and depleted negative charge at position N(1) (*nq* = −0.05), which is consistent with the Lewis resonance structure shown in Figure 8. Further analysis shows a slightly higher electrophilicity of position C(1′) (a total CH *nq* = 0.06) than C(3′) with a total CH *nq* of 0.03.

**Figure 9:**
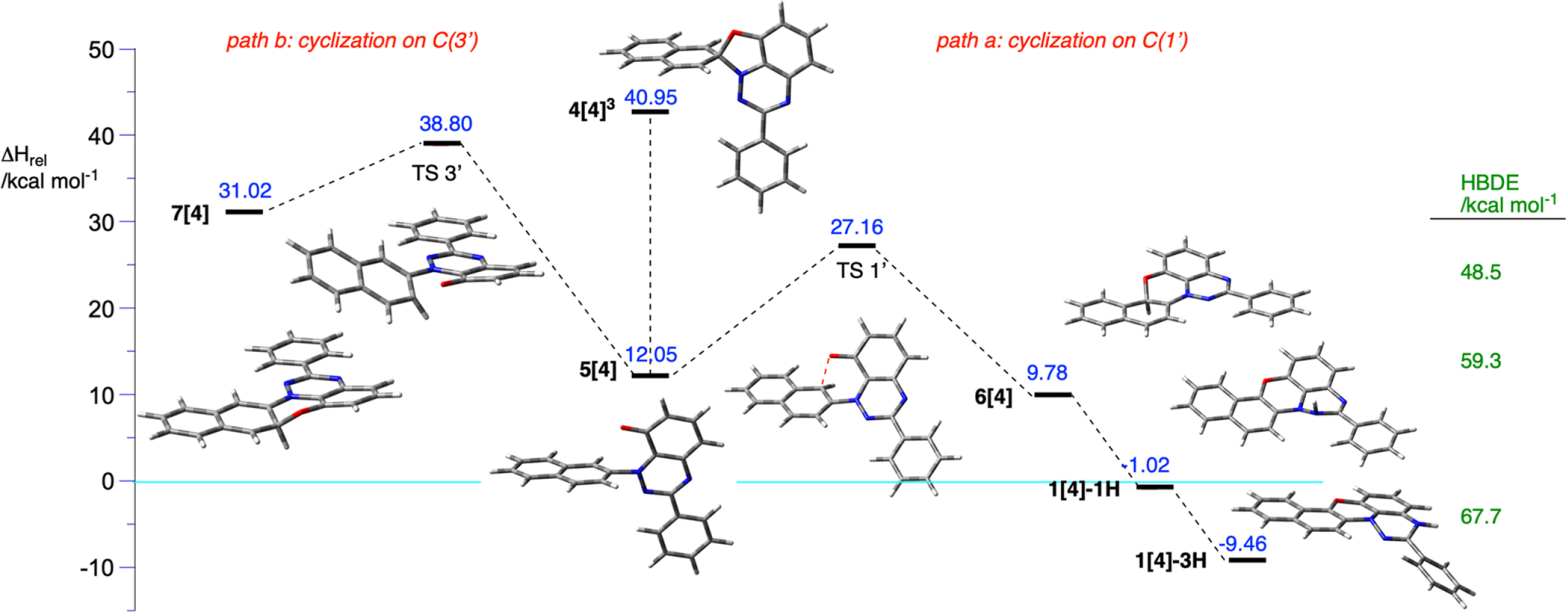
Energies of intermediates and transition states relative to that of **2[4]** in the ground state [Δ*H* = 0 kcal mol^–1^ (blue line)] for the proposed mechanism of photocyclization of **2[4]** obtained at the CAM-B3LYP/6-311G(d,p) level of theory in the EtOAc dielectric medium. Homolytic bond dissociation energies (HBDEs) calculated for selected species are shown on the right (green). For details, see the text.

The oxygen atom in zwitterion **5[4]** can move to position C(1′) (path a in Figure 8) or C(3′) (path b), giving rise to dipolar intermediate **6[4]** or **7[4]**, respectively. Analysis of the Lewis structures indicates that the latter product has one fewer Clar’s sextet, which renders it significantly less stable than its C(1′) isomer **6[4]** (via DFT, Δ*H* = 21.24 kcal mol^–1^). Intermediate **6[4]** is moderately more thermodynamically stable than zwitterion **5[4]** (Δ*H* = −2.27 kcal mol^–1^), and considering a relatively low activation free energy (Δ*G*^⧧^_298_ = 17.33 kcal mol^–1^), both compounds **5[4]** and **6[4]** can be in thermal equilibrium at ambient temperature (Figure 9). In contrast, the calculated activation free energy for the formation of isomeric **7[4]** is 29.04 kcal mol^–1^, which renders it inaccessible under the reaction conditions. Further stabilization of intermediate **6[4]** and its removal from the equilibrium with **5[4]** occur through tautomerization by formal H^+^ migration from C(1′) either to N(1) (**1[4]-1H**) and then to N(3) (**1[4]-H**) or directly to N(3) in the most thermodynamically stable tautomer, **1[4]-H**. The tautomerization to **1[4]-H** with simultaneous recovery of the second Clar’s sextet renders this process exothermic by 19.24 kcal mol^–1^, while the entire phototransformation of **2[4]** to **1[4]-H** is moderately exothermic by 9.46 kcal mol^–1^ (Figure 9).

Subsequent oxidation of **1[4]-H**, presumedly with molecular oxygen present in the solution, leads to experimentally observed radical **1[4]**. Alternatively, oxidative removal of the C(1′) hydrogen atom in **6[4]** and formation of **1[4]** may take place without prior tautomerization. The calculated homolytic bond dissociation enthalpy (HBDE) for the three tautomeric forms **6[4]**, **1[4]-1H**, and **1[4]-H** using the experimental value for phenoxazine^31^ as the reference shows a low HBDE for the first tautomer (48.5 kcal mol^–1^), related to the recovery of the second Clar’s sextet, and 59.3 and 67.7 kcal mol^–1^ for last two NH tautomers. The last value for **1[4]-H** is in the range expected for Blatter radical derivatives.^32^ A summary of the proposed mechanism for the photocyclization of **2[4]** is shown in Figure 9.

## Discussion

The mechanism proposed for the photocyclization of **2[4]** has four key elements: (i) the (n, π*) character of the lowest excited state localized on the BT fragment, which, according to the Kasha rule, is responsible for the subsequent transformations (it is believed that the T_1_ state is key to the observed photochemistry); (ii) the presence of at least one higher-energy occupied MO localized on the aryloxy substituent; (iii) the proximity of the BT and aryloxy fragments for an efficient intramolecular electron transfer; and (iv) the high positive charge density (high electrophilicity) localized on the aryl carbon atom connected to the oxygen atom.

**Figure 10:**
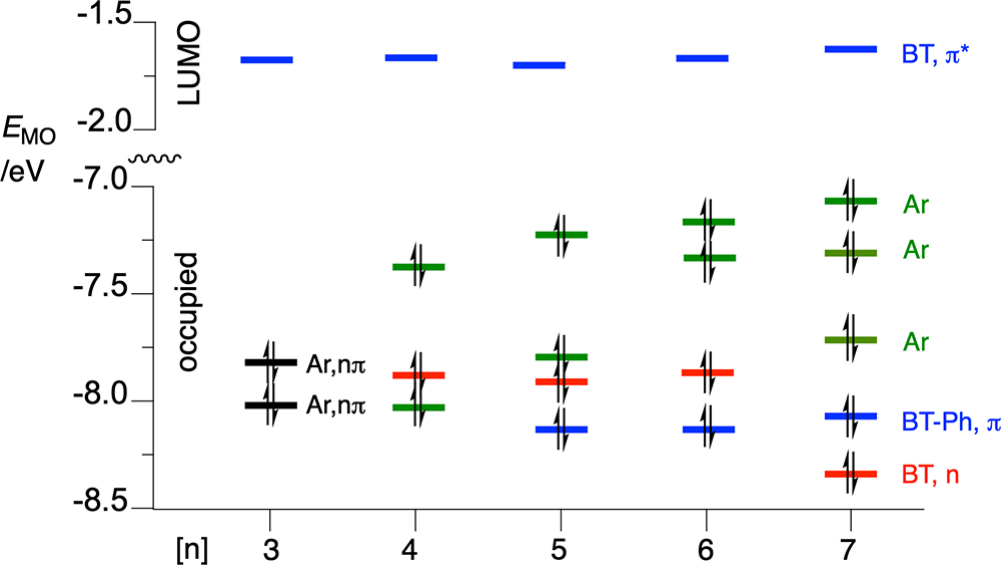
Energy and assignment of MOs for precursors **2[*n*]** in the T_1_ state geometry in an EtOAc dielectric medium [CAM-B3LYP/6311(d,p)]. Colors indicate the type and localization of the MO: blue for the π MO on the heterocycle (BT, π) and the heterocycle with C(3)-Ph (BT-Ph, π), red for the n MO on the heterocycle (BT, n), green for π aryl (Ar), and black for mixed n BT and π Ar.

In the first approximation, these requirements can be assessed using standard DFT calculations for the substrate undergoing photocyclization: determination of the nature of the T_1_ state, order and localization of the occupied high MOs, and charge distribution of radical ions at the substrate’s GS geometry. Such an analysis for other members of series **2[*n*]** demonstrates that all exhibit electronic structural features required for photocyclization with Smiles rearrangement (Figure 10). Thus, in all compounds, the T_1_ state is the ^3^(n, π*) state localized on the BT fragment, and all, except for **2[3]**, have at least one π aryl MO above the n MO (Figure 10). In the case of **2[3]**, the T_1_ state involves the HOMO and HOMO–1, which encompass both the nitrogen atom lone pairs of the BT and the π system of the PhO group, while in higher members of the series, the two electronic systems constitute separate MOs. This electronic structure of **2[3]** permits a direct photoinduced charge polarization necessary for the formation of the spirooxazole and Smiles rearranged products, while in higher homologues with the two electronic systems disconnected, an intramolecular CT is needed. This charge polarization or formation of zwitterions is supported by the observed higher efficiency and higher isolated yields of the photocyclized products in polar media, such as in EtOAc and EtOH versus CH_2_Cl_2_.^24,25^

**Figure 11:**
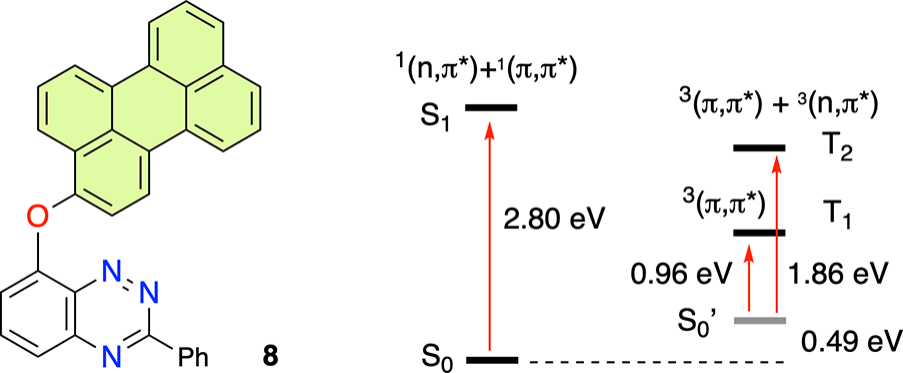
Partial Jablonski diagram for perylene derivative **8** based on TD-DFT analysis.

Further calculations indicate that photocyclization of other 8-aryloxybenzo[*e*][1,2,4]triazinyl precursors conforms to the proposed mechanism, which requires revisiting the structural assignments of those products and will be reported elsewhere. Detailed DFT analysis of perylenoxy derivative **8**, which is inert^24^ in the photocyclization process, demonstrates that its T_1_ state completely lacks the (n, π*) character localized on the BT unit necessary for zwitterion formation (Figure 11). Instead, T_1_ and T_2_ are the ^3^(π, π*) states solely localized on the perylene fragment.

The proposed mechanism is significantly different from those previously considered for the photo-Smiles rearrangements shown in Figure 1. Thus, none of those mechanisms involve both intramolecular radical ion pair photogeneration and oxazole formation in a polar addition process, which is postulated for cyclization of **2[*n*]**. Also, none of the previously reported processes lead to cyclic products. Therefore, photocyclization of **2[*n*]** appears to be unique among a broad and rich spectrum of Smiles rearrangements^2^ and related aryl transfer reactions.^21^

**Scheme 2:**
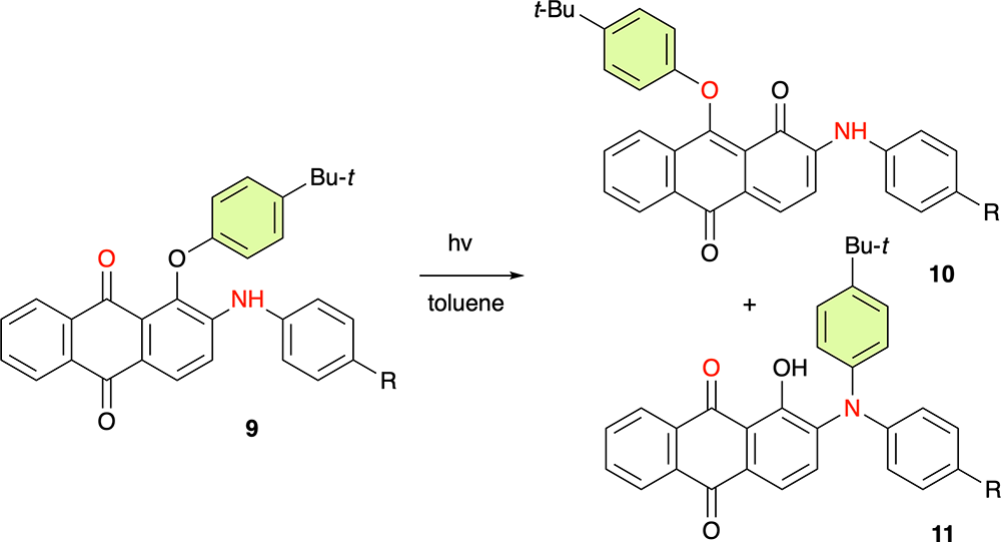
Photorearrangement of an Anthraquinone Derivative^33^

A process that may be mechanistically closest to the currently reported photocyclization is a photorearrangement of aryloxyantraquinones **9** (Scheme 2).^33^ Although the authors did not discuss the mechanism, it can be postulated that an open-shell zwitterion, analogous to **2[4]**^**Z**^, is formed by SET between the aryloxy group and the excited quinone fragment, which subsequently undergoes intramolecular nucleophilic addition of either [C=O]^−•^ or NH, formation of the spirooxazole, and finally ring opening. This proposal is consistent with the reported^33^ formation of two competing products, **10** and **11** (Scheme 2), both presumably arising from intramolecular addition of a nucleophile (C=O radical anion and NHAr, respectively) to the electrophilic center of the aryloxy radical cation.

## Summary and Conclusions

Mechanistic investigation revealed that photocyclization of 8-aryloxybenzo[*e*][1,2,4]triazines **2[*n*]** and formation of **1[*n*]-H** proceed through a novel photo-Smiles rearrangement process, which involves the formation of a radical zwitterion through an (n, π*) state localized on the BT fragment, followed by an intramolecular SET process. The subsequent polar cyclization of the resulting radical zwitterion yields a spirooxazole intermediate, which selectively transforms into *leuco***1[*n*]-H** (or its tautomers) as a direct precursor to radical **1[*n*]**. The migration of the oxygen atom in the rearrangement process is fully regioselective, as confirmed with correlation ^1^H–^1^H NMR analysis, and governed by the preservation of the Clar’s sextets in the products.

The key step in this novel cyclization mechanism, the photogeneration of the radical zwitterion capable of polar cyclization, is believed to be general for photocyclization of 8-substituted benzo[*e*][1,2,4]triazines. This includes other aryloxy derivatives that undergo Smiles rearrangement and also 8-carbazole and 8-aryl derivatives that photocyclize without Smiles rearrangement. These reactions and analyses will be described in due course. The proposed mechanism is consistent with the observed solvent effects on photocyclization efficiency and the complete photochemical inertness of the 8-perylenoxy derivative.

Overall, the described results provide the first mechanistic understanding of photogeneration of π-extended planar Blatter radicals and offer a predictive tool for the design of other substrates, leading to novel stable radicals.

## Computational Details

Quantum-mechanical calculations were carried out using the Gaussian 16 suite of programs.^34^ Geometry optimizations were undertaken at the (U)CAM-B3LYP/6-311G(d,p) and B3LYP/6-31G(2d,p) levels of theory in a solvent dielectric medium using tight convergence criteria. Solvent effects were implemented with the PCM model^35^ and the SCRF keyword.

Mechanistic analysis of the photocyclization of **2[4]** was performed at the (U)CAM-B3LYP/6-311G(d,p) level of theory in EtOAc dielectric medium requested with the SCRF (solvent = ethylethanoate) keyword. Electronic excitation energies in EtOAc dielectric medium were obtained for **2[4]** and other 8-aryloxybenzo[*e*][1,2,4]triazines at the CAM-B3LYP/6-311G(d,p)//CAM-B3LYP/6-311G(d,p) level of theory using the time-dependent^36^ DFT method supplied in Gaussian 16 and TD = (singlets, root = 1, NStates = 12) or TD=(triplets, root = 1, NStates = 12) keyword.

NMR isotropic shielding constants for the *leuco* derivatives were calculated using the B3LYP/6-311G(2d,p)//B3LYP/6-31G(2d,p) method in DMSO dielectric medium requested with the SCRF (solvent = DiMethylSulfoxide) keyword.

## Data Availability

The data underlying this study are available in the published article and its Supporting Information.

## Supporting Information Available

Details of the synthesis and characterization of compounds, NMR spectra, and computational details (PDF)
FAIR data, including the primary NMR FID files, for compounds **1[4]-H**–**1[7]-H** (ZIP)

## References

[ref1] Levy A. A.; Rains H. C.; Smiles S. The rearrangement of hydroxy-sulphones Part I. J. Chem. Soc. 1931, 0, 3264–3269. 10.1039/JR9310003264.

[ref2] Holden C. M.; Greaney M. F. Modern aspects of the Smiles rearrangement. Chem. - Eur. J. 2017, 23, 8992–9008. 10.1002/chem.201700353. 28401655

[ref3] Kumar K. S.; Gugulothu K.; Reddy S. R.; Venkateswarlu K. A critical review on recent advances in base-assisted Smiles rearrangement. Curr. Org. Chem. 2022, 26, 1303–1310. 10.2174/1385272826666220509143140.

[ref4] Truce W. E.; Kreider E. M.; Brand W. W. The Smiles and related rearrangements of aromatic systems; Wiley: Hoboken, NJ, 1970.

[ref5] Snape T. J. A truce on the Smiles rearrangement: Revisiting an old reaction—the Truce–Smiles rearrangement. Chem. Soc. Rev. 2008, 37, 2452–2458. 10.1039/b808960d. 18949118

[ref6] Henderson A. R. P.; Kosowan J. R.; Wood T. E. The Truce–Smiles rearrangement and related reactions: a review. Can. J. Chem. 2017, 95, 483–504. 10.1139/cjc-2016-0594.

[ref7] Liu H.; Jiang X. Transfer of sulfur: From simple to diverse. Chem. - Asian J. 2013, 8, 2546–2563. 10.1002/asia.201300636. 23846983

[ref8] El Kaïm L.; Grimaud L. The Ugi–Smiles and Passerini–Smiles couplings: A story about phenols in isocyanide-based multicomponent reactions. Eur. J. Org. Chem. 2014, 2014, 7749–7762. 10.1002/ejoc.201402783.

[ref9] Pluta K.; Morak-Młodawska B.; Jeleń M. The Smiles rearrangement in the syntheses of azaphenothiazines. Part II. The review of the various types of phenyl azinyl and diazinyl sulfides undergoing this rearrangement. J. Mol. Struct. 2020, 1216, 128320 10.1016/j.molstruc.2020.128320.

[ref10] Whalley D. M.; Greaney M. F. Recent advances in the Smiles rearrangement: New opportunities for arylation. Synthesis 2022, 54, 1908–1918. 10.1055/a-1710-6289.

[ref11] Yang B.; Tan X.; Guo R.; Chen S.; Zhang Z.; Chu X.; Xie C.; Zhang D.; Ma C. Transition metal-free one-pot synthesis of fused 1,4-thiazepin-5(4*H*)-ones and theoretical study of the S–N type Smiles rearrangement process. J. Org. Chem. 2014, 79, 8040–8048. 10.1021/jo5011729. 25101862

[ref12] Cheron N.; Jacquemin D.; Fleurat-Lessard P. A qualitative failure of B3LYP for textbook organic reactions. Phys. Chem. Chem. Phys. 2012, 14, 7170–7175. 10.1039/c2cp40438a. 22491187

[ref13] Douglas J. J.; Albright H.; Sevrin M. J.; Cole K. P.; Stephenson C. R. J. A visible-light-mediated radical Smiles rearrangement and its application to the synthesis of a difluoro-substituted spirocyclic ORL-1 antagonist. Angew. Chem., Int. Ed. 2015, 54, 14898–14902. 10.1002/anie.201507369. PMC4725294 26474077

[ref14] Wu X.; Ma Z.; Feng T.; Zhu C. Radical-mediated rearrangements: past, present, and future. Chem. Soc. Rev. 2021, 50, 11577–11613. 10.1039/D1CS00529D. 34661216

[ref15] Allart-Simon I.; Gérard S.; Sapi J. Radical Smiles Rearrangement: An Update. Molecules 2016, 21, 878 10.3390/molecules21070878. 27399654 PMC6273782

[ref16] Matsui K.; Maeno N.; Suzuki S.; Shizuka H.; Morita T. Photo-Smiles rearrangements. Tetrahedron Lett. 1970, 11, 1467–1469. 10.1016/S0040-4039(01)97997-3.

[ref17] Mutai K.; Kanno S.; Kobayashi K. The photo-Smiles rearrangement. Tetrahedron Lett. 1978, 19, 1273–1276. 10.1016/0040-4039(78)80104-X.

[ref18] Yokoyama K.; Nakagaki R.; Nakamura J.; Mutai K.; Nagakura S. Spectroscopic and kinetic study of an intramolecular aromatic nucleophilic photosubstitution. Reaction mechanism of a photo-Smiles rearrangement. Bul. Chem. Soc. Jpn. 1980, 53, 2472–2475. 10.1246/bcsj.53.2472.

[ref19] Wubbels G. G.; Sevetson B. R.; Sanders H. Competitive catalysis and quenching by amines of photo-Smiles rearrangement as evidence for a zwitterionic triplet as the proton-donating intermediate. J. Am. Chem. Soc. 1989, 111, 1018–1022. 10.1021/ja00185a034.

[ref20] Wubbels G. G.; Cotter W. D.; Sanders H.; Pope C. Bronsted catalysis law plots for heterolytic, general base-catalyzed Smiles photorearrangement. J. Org. Chem. 1995, 60, 2960–2961. 10.1021/jo00115a005.

[ref21] Allen A. R.; Noten E. A.; Stephenson C. R. J. Aryl transfer strategies mediated by photoinduced electron transfer. Chem. Rev. 2022, 122, 2695–2751. 10.1021/acs.chemrev.1c00388. 34672526 PMC9272681

[ref22] Lawson C. A.; Dominey A. P.; Williams G. D.; Murphy J. A. Visible light-mediated Smiles rearrangements and annulations of non-activated aromatics. Chem. Commun. 2020, 56, 11445–11448. 10.1039/D0CC04666C. 32852011

[ref23] Bartos P.; Young V. G. Jr; Kaszyński P. Ring-fused 1,4-dihydro[1,2,4]triazin-4-yls through photocyclization. Org. Lett. 2020, 22, 3835–3840. 10.1021/acs.orglett.0c01074. 32330048

[ref24] Zissimou G. A.; Bartos P.; Pietrzak A.; Kaszyński P. “Upper” ring expansion of the planar Blatter radical via photocyclization: Limitations and impact on the electronic structure. J. Org. Chem. 2022, 87, 4829–4837. 10.1021/acs.joc.2c00178. 35290052

[ref25] Singh H. K.; Bodzioch A.; Pietrzak A.; Kaszyński P. π-Curved Blatter radicals: Blatter helicenes. Chem. Commun. 2025, 61, 496–499. 10.1039/D4CC05704J. 39641166

[ref26] Bax A.; Davis D. G. MLEV-17-based two-dimensional homonuclear magnetization transfer spectroscop. J. Magn. Reson. 1985, 65, 355–360. 10.1016/0022-2364(85)90018-6.

[ref27] Hwang T.-L.; Shaka A. J. Cross relaxation without TOCSY: transverse rotating-frame Overhauser effect spectroscopy. J. Am. Chem. Soc. 1992, 114, 3157–3159. 10.1021/ja00034a083.

[ref28] For details, see the Supporting Information.

[ref29] El-Sayed M. A. Triplet state. Its radiative and nonradiative properties. Acc. Chem. Res. 1968, 1, 8–16. 10.1021/ar50001a002.

[ref30] Scott G. W.; Talley L. D.; Anderson R. W. Jr. Excited state absorption spectra and intersystem crossing kinetics in diazanaphthalenes. J. Chem. Phys. 1980, 72, 5002–5013. 10.1063/1.439788.

[ref31] Lucarini M.; Pedrielli P.; Pedulli G. F.; Valgimigli L.; Gigmes D.; Tordo P. Bond dissociation energies of the N-H bond and rate constants for the reaction with alkyl, alkoxyl, and peroxyl radicals of phenothiazines and related compounds. J. Am. Chem. Soc. 1999, 121, 11546–11553. 10.1021/ja992904u.

[ref32] Bartos P.; Hande A. A.; Pietrzak A.; Chrostowska A.; Kaszyński P. Substituent effects on the electronic structure of the flat Blatter radical: Correlation analysis of experimental and computational data. New J. Chem. 2021, 45, 22876–22887. 10.1039/D1NJ05137G.

[ref33] Mainagashev I. Y.; Klimenko L. S.; Gritsan N. P. Photochemical and thermal rearrangements of 2-arylamino-1- (4-tert-butylphenoxy)-9,10-anthraquinones. Russ. Chem. Bull. 1998, 47, 2437–2440. 10.1007/BF02641550.

[ref34] Frisch M. J.; Trucks G. W.; Schlegel H. B.; Scuseria G. E.; Robb M. A.; Cheeseman J. R.; Scalmani G.; Barone V.; Petersson G. A.; Nakatsuji H.; Li X.; Caricato M.; Marenich A. V.; Bloino J.; Janesko B. G.; Gomperts R.; Mennucci B.; Hratchian H. P.; Ortiz J. V.; Izmaylov A. F.; Sonnenberg J. L.; Williams-Young D.; Ding F.; Lipparini F.; Egidi F.; Goings J.; Peng B.; Petrone A.; Henderson T.; Ranasinghe D.; Zakrzewski V. G.; Gao J.; Rega N.; Zheng G.; Liang W.; Hada M.; Ehara M.; Toyota K.; Fukuda R.; Hasegawa J.; Ishida M.; Nakajima T.; Honda Y.; Kitao O.; Nakai H.; Vreven T.; Throssell K.; Montgomery J. A. Jr.; Peralta J. E.; Ogliaro F.; Bearpark M. J.; Heyd J. J.; Brothers E. N.; Kudin K. N.; Staroverov V. N.; Keith T. A.; Kobayashi R.; Normand J.; Raghavachari K.; Rendell A. P.; Burant J. C.; Iyengar S. S.; Tomasi J.; Cossi M.; Millam J. M.; Klene M.; Adamo C.; Cammi R.; Ochterski J. W.; Martin R. L.; Morokuma K.; Farkas O.; Foresman J. B.; Fox D. J. Gaussian 16; Gaussian, Inc.: Wallingford, CT, 2016.

[ref35] Cossi M.; Scalmani G.; Rega N.; Barone V. New developments in the polarizable continuum model for quantum mechanical and classical calculations on molecules in solution. J. Chem. Phys. 2002, 117, 43–54. 10.1063/1.1480445.

[ref36] Stratmann R. E.; Scuseria G. E.; Frisch M. J. An efficient implementation of time-dependent density-functional theory for the calculation of excitation energies of large molecules. J. Chem. Phys. 1998, 109, 8218–8224. 10.1063/1.477483.

