# Supplementary material for: A Photo-Smiles Rearrangement: Mechanistic Investigation of the Formation of Blatter Radical Helicenes
Source: J Org Chem. 2025 Feb 5;90(6):2386–92. doi: 10.1021/acs.joc.4c02893 (PMC11833871; doi:10.1021/acs.joc.4c02893)
Supplement: Supplementary file 1 — jo4c02893_si_001.pdf [file jo4c02893_si_001.pdf]

A photo-Smiles rearrangement: Mechanistic investigation of the formation of Blatter radical helicenes

Hemant K. Singh,[§] Sławomir Kaźmierski,[§] and Piotr Kaszyński*[§‡#]

[§] Centre of Molecular and Macromolecular Studies, Polish Academy of Sciences, 90-363 Łódź, Poland

[‡] Faculty of Chemistry, University of Łódź, 91-403 Łódź, Poland

[#] Department of Chemistry, Middle Tennessee State University, Murfreesboro, TN 37130, USA

Table of contents **Page**

1. Experimental Section ……S2

2. 1D ^{1}H NMR spectra of **1[n]-H** ……S4

3. ^{1}H–^{1}H NMR correlation spectra of **1[n]-H** ……S6

4. Computational details ……S15

a) geometry optimization of *leuco* forms **1[n]-H** ...S15
b) ^{1}H NMR chemical shift calculations for **1[n]-H** ...S15
c) mechanistic investigation of photocyclization of **2[4]** ...S17
d) partial output from TD-DFT calculation for **2[4]** ...S17
e) N–H bond dissociation energy ...S19

5. Archive for DFT calculations ……S21

6. References ……S31

1. Experimental Section

^1H NMR spectra were recorded in DMSO-d_6, containing a drop of CD_2Cl_2 and D_2O on AV III 500 MHz Bruker NMR spectrometer. Chemical shifts are reported in δ ppm relative to DMSO-d_6 residual peak at δ_{1H} = 2.50 ppm.

Signals at 5.72, 4.75 and below 4.5 ppm in each spectrum are due to the residual protonated solvents (CD_2Cl_2, D_2O, DMSO-d_6) and ascorbic acid

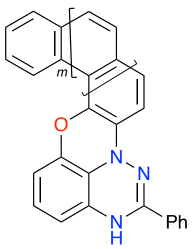

Generation of *leuco* 1[n]-H by reduction of radicals 1[n]. A general procedure. Radical **1[n]** (typically 2 mg, 1 equiv.) and ascorbic acid (1.2 equiv.) was taken in a 5 mL of RB flask and added D_2O (1 drop), CD_2Cl_2 (2 drops) and DMSO-d_6 (0.4 mL). After 15 min of stirring, sample the solution was placed in an NMR tube and ^1H NMR spectrum was recorded. In some cases, complete reduction takes up to 3 hrs.

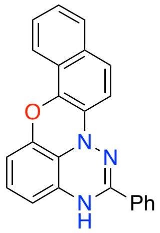

1[4]-H. ^1H NMR (500 MHz, DMSO-d_6) δ 7.92 (dd, J_1 = 8.0 Hz, J_2 = 1.0 Hz, 1H), 7.87 (dd, J_1 = 7.5 Hz, J_2 = 1.5 Hz, 2H), 7.79 (d, J = 8.0 Hz, 1H), 7.62 (q, J = 9.0 Hz, 2H), 7.52-7.46 (m, 4H), 7.37-7.34 (m, 1H), 6.79 (t, J = 8.0 Hz, 1H), 6.55 (dd, J_1 = 8.0 Hz, J_2 = 1.0 Hz, 1H), 6.41 (dd, J_1 = 8.0 Hz, J_2 = 1.5 Hz, 1H).

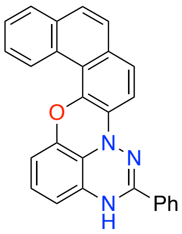

1[5]-H. ^1H NMR (500 MHz, DMSO-d_6) δ 9.44 (d, J = 7.5 Hz, 1H), 7.91 (dd, J_1 = 7.5 Hz, J_2 = 2.0 Hz, 1H), 7.88 (dd, J_1 = 7.5 Hz, J_2 = 1.5 Hz, 2H), 7.67-7.60 (m, 6H), 7.53-7.48 (m, 3H), 6.78 (t, J = 8.0 Hz, 1H), 6.73 (dd, J_1 = 8.5 Hz, J_2 = 1.5 Hz, 1H), 6.39 (dd, J_1 = 7.5 Hz, J_2 = 1.5 Hz, 1H).

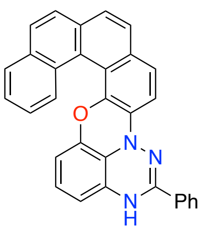

1[6]-H. ^1H NMR (500 MHz, DMSO-d_6) δ 8.20 (d, J = 9.0 Hz, 1H), 8.04 (dd, J_1 = 7.0 Hz, J_2 = 1.5 Hz, 2H), 7.91-7.89 (m, 3H), 7.83 (d, J = 8.5 Hz, 1H), 7.78 (q, J = 9.0 Hz, 2H), 7.70 (d, J = 8.5 Hz, 1H), 7.61-7.58 (m, 1H), 7.54-7.50 (m, 4H), 6.70 (t, J = 8.0 Hz, 1H), 6.39 (dd, J_1 = 7.5 Hz, J_2 = 1.0 Hz, 1H), 5.90 (dd, J_1 = 8.0 Hz, J_2 = 1.0 Hz, 1H).

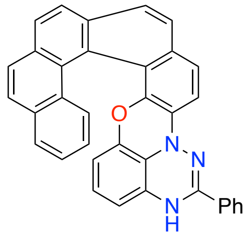

1[7]-H. ^{1}H NMR (500 MHz, DMSO-d_6) δ 8,12-8.05 (m, 3H), 8.03 (d, J = 3.5 Hz, 2H), 8.01 (t, J = 3.0 Hz, 1H), 7.91 (d, J = 8.0 Hz, 1H), 7.87 (dd, J_1 = 7.5 Hz, J_2 = 1.5 Hz, 2H), 7.81 (d, J = 4.5 Hz, 1H), 7.79 (d, J = 4.5 Hz, 1H), 7.72 (d, J = 9.0 Hz, 1H), 7.52-7.48 (m, 3H), 7.39 (ddd, J_1 = 8.0 Hz, J_2 = 7.0 Hz, J_3 = 1.5 Hz, 1H), 7.30 (ddd, J_1 = 8.5 Hz, J_2 = 7.0 Hz, J_3 = 1.5 Hz, 1H), 6.24 (t, J = 8.0 Hz, 1H), 6.13 (dd, J_1 = 8.0 Hz, J_2 = 1.0 Hz, 1H), 4.53 (dd, J_1 = 8.0 Hz, J_2 = 1.5 Hz, 1H).

2. 1D ^{1}H NMR spectra of 1[n]-H

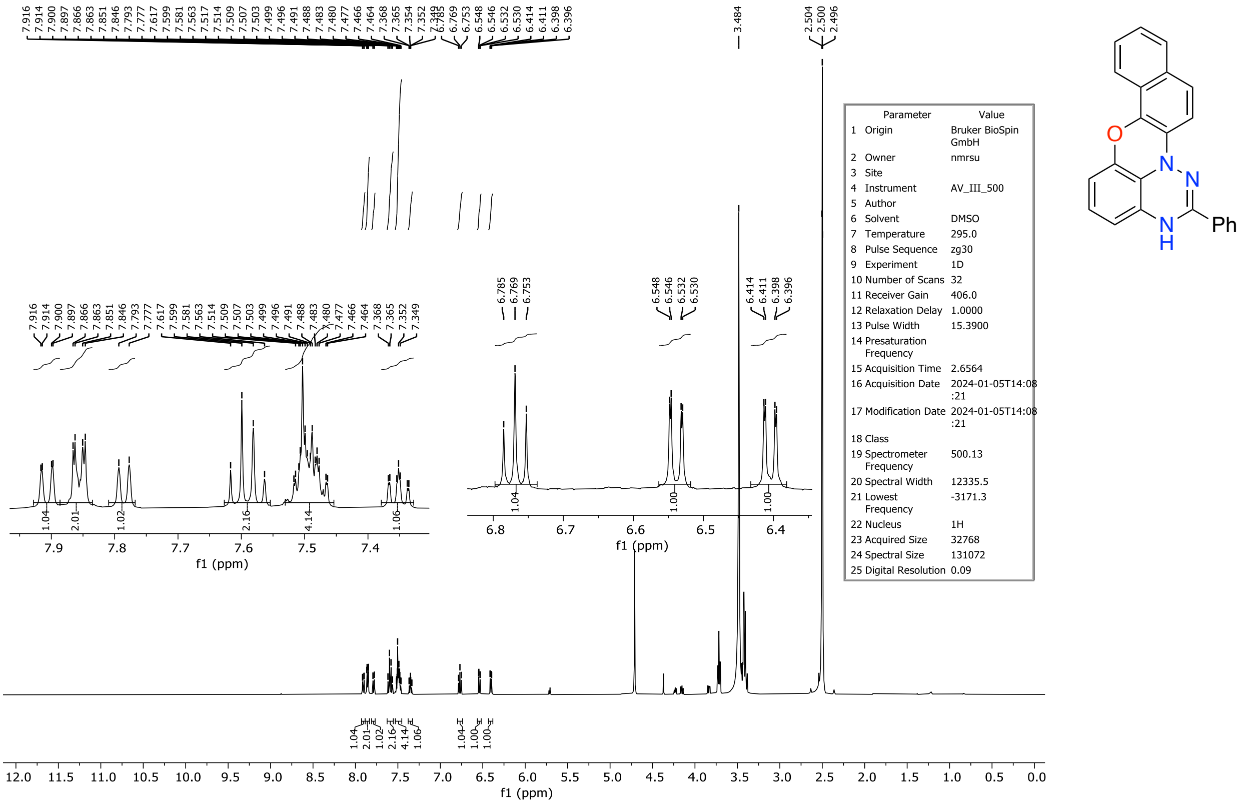

Figure S1. ^{1}H NMR of freshly generated **1[4]-H** recorded in DMSO-d_6 containing a drop of CD_2Cl_2 and D_2O at 500 MHz.

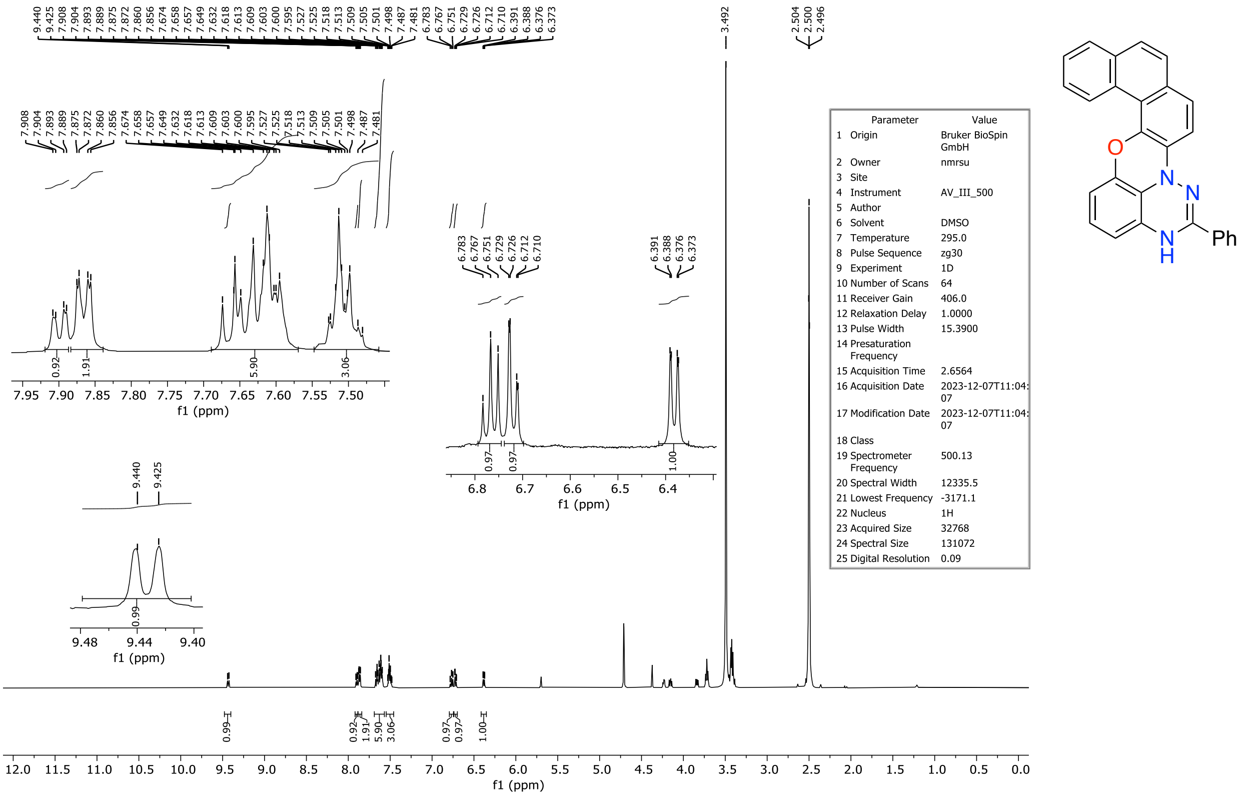

Figure S2. ^{1}H NMR of freshly generated **1[5]-H** recorded in DMSO-d_6 containing a drop of CD_2Cl_2 and D_2O at 500 MHz.

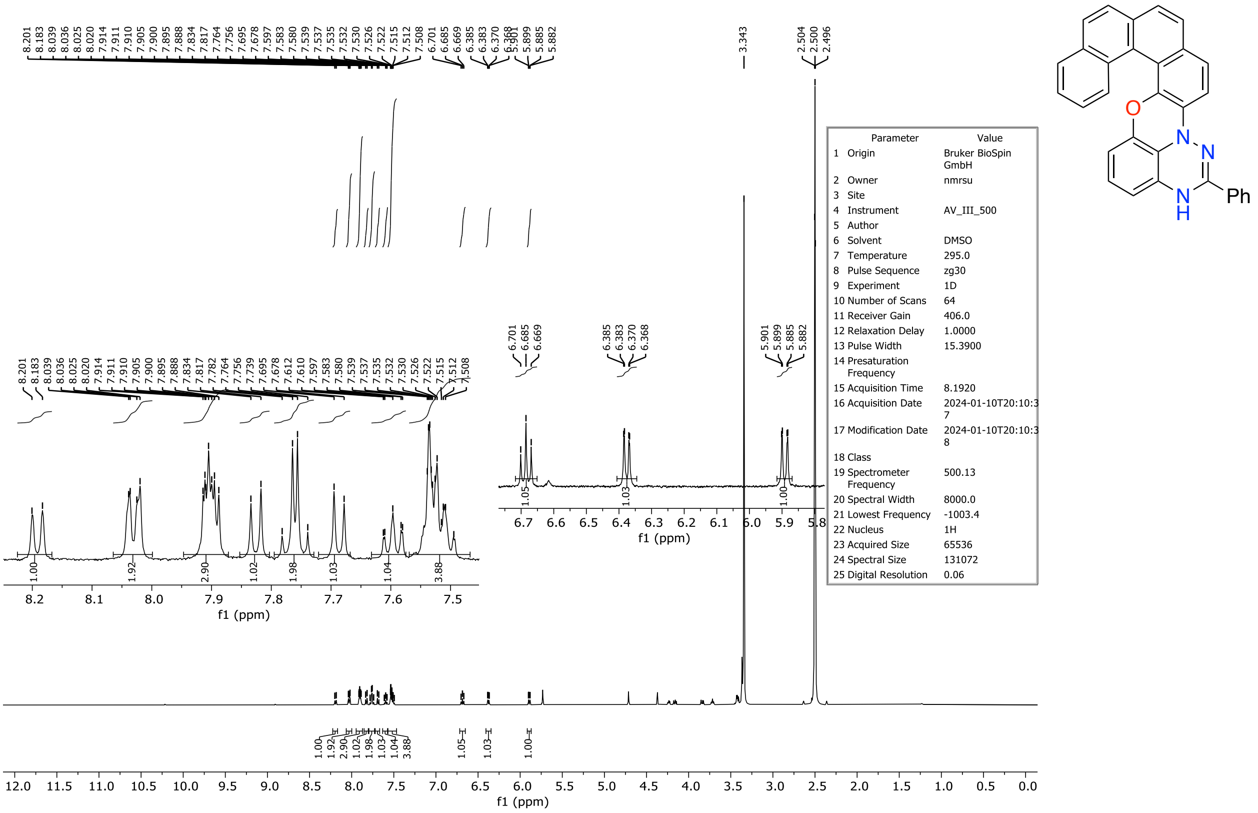

Figure S3. ^{1}H NMR of freshly generated **1[6]-H** recorded in DMSO-d_6 containing a drop of CD_2Cl_2 and D_2O at 500 MHz.

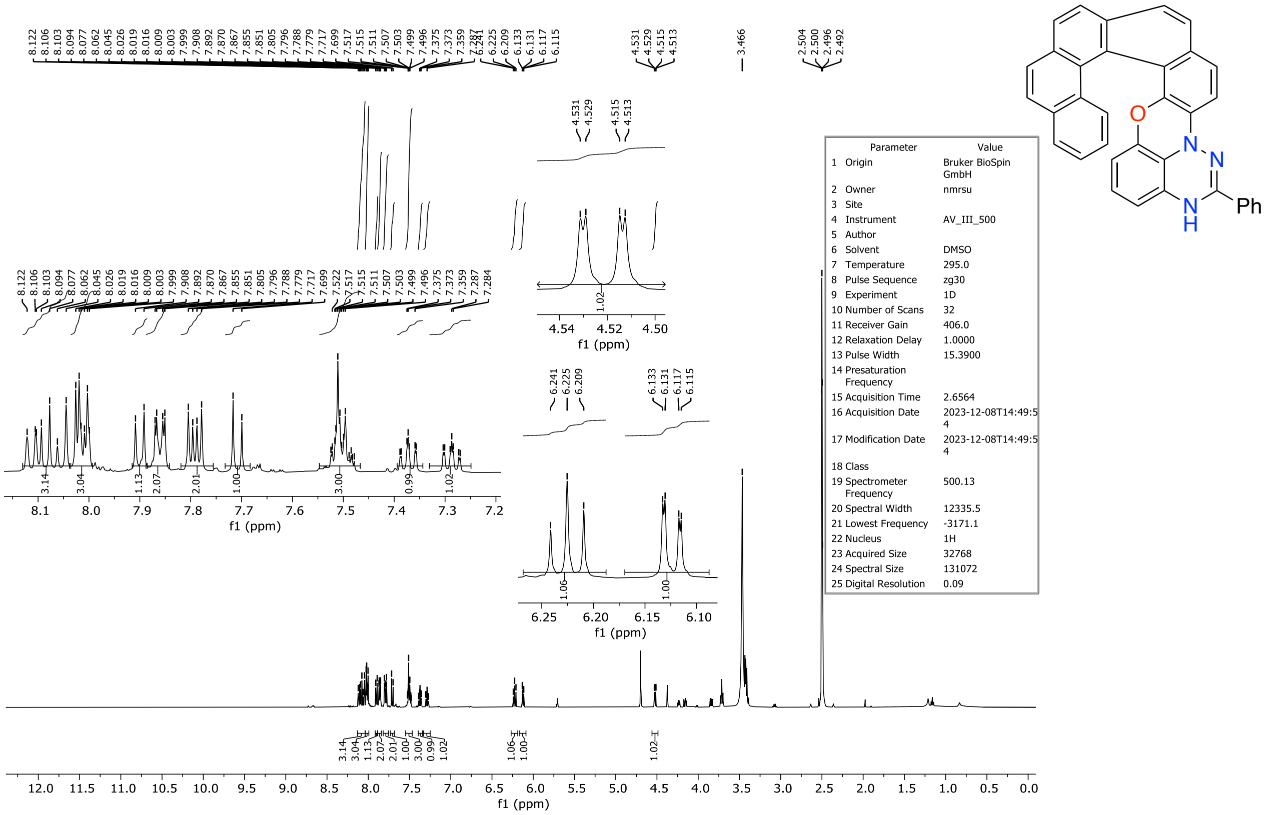

Figure S4. ^{1}H NMR of freshly generated **1[7]-H** recorded in DMSO-d_6 containing a drop of CD_2Cl_2 and D_2O at 500 MHz.

3. ^{1}H–^{1}H NMR correlation spectra of **1[n]-H**

All measurements were performed on Bruker Avance III 500 spectrometer (Bruker BioSpin, Rheinstetten, Germany), operating at frequency of 500.13 MHz for ^{1}H and equipped with GAB/2 gradient unit capable to produce B_0 gradients with maximum strength of 50 G/cm. Automated tuned and matched (ATMA) 5 mm triple channel TBO (BB/H-F/D) probe head with actively shielded Z-gradients coil was utilized. During all measurements, the temperature was controlled and stabilized with BCU 05 cooling unit managed by BVT3200 variable temperature unit. All spectra of **1[n]-H** were recorded in 5 mm NMR tubes using a mixture of deuterated DMSO, CD_2Cl_2 and D_2O solvents. For chemical shift calibration the residual signal of DMSO-d_6 was used (δ_{1H} = 2.49 ppm). For each sample the temperature was stabilized at 295 K for at least 5 minutes and the ^{1}H $\pi/2$ pulse length was checked and corrected before data accumulation. All spectra were acquired, processed and plotted using TopSpin 3.5(pl6) program running on PC computer under Windows 7 Professional.

For 1D ^{1}H spectra 64 scans were accumulated per FID of 64K data points with 1s relaxation delay (D1) and spectral width was set to 12000 Hz (10 ppm) results in 2.64 s of acquisition time (AQ). Original pulse program zg30 was used. FIDs were zero-filled twice and apodized with LB function of 0.3Hz prior to Fourier transformation.

For 2D COSY, TOCSY and ROESY spectra parameters were as follow: spectra were acquired in 4096 x 512 (F2xF1) data points matrix with 16 (COSY) or 32 (TOCSY, ROESY) scans for each experiment and 32 dummy scans and relaxation delay (D1) of 1.5 s. The spectral width was 5000 Hz (10 ppm) in both dimensions. Prior to Fourier transformation into a final 2048 x 2048 data points matrix, FIDs were apodised with QSINE (2) function in F2 and F1 dimensions. Automatic baseline correction in both dimensions was applied on final 2D spectra. Neither linear prediction nor summarization was applied. Original Bruker pulse programs ***cosygpppqf***, ***mlevph*** and ***roesyphpp.2*** were utilized for COSY, TOCSY[1] and ROESY[2] respectively. TOCSY was run with mixing time (D9) of 120 ms and for ROESY the spin lock time (P15) was set to 350 ms.

The resulting TOCSY and ROESY spectra with indicated structural assignments are shown in Figures S5–S12. A summary of structural assignment of key ^{1}H NMR signals is shown in Table S1.

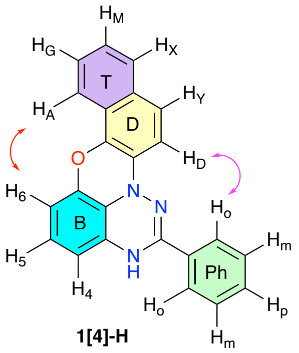

1[4]-H

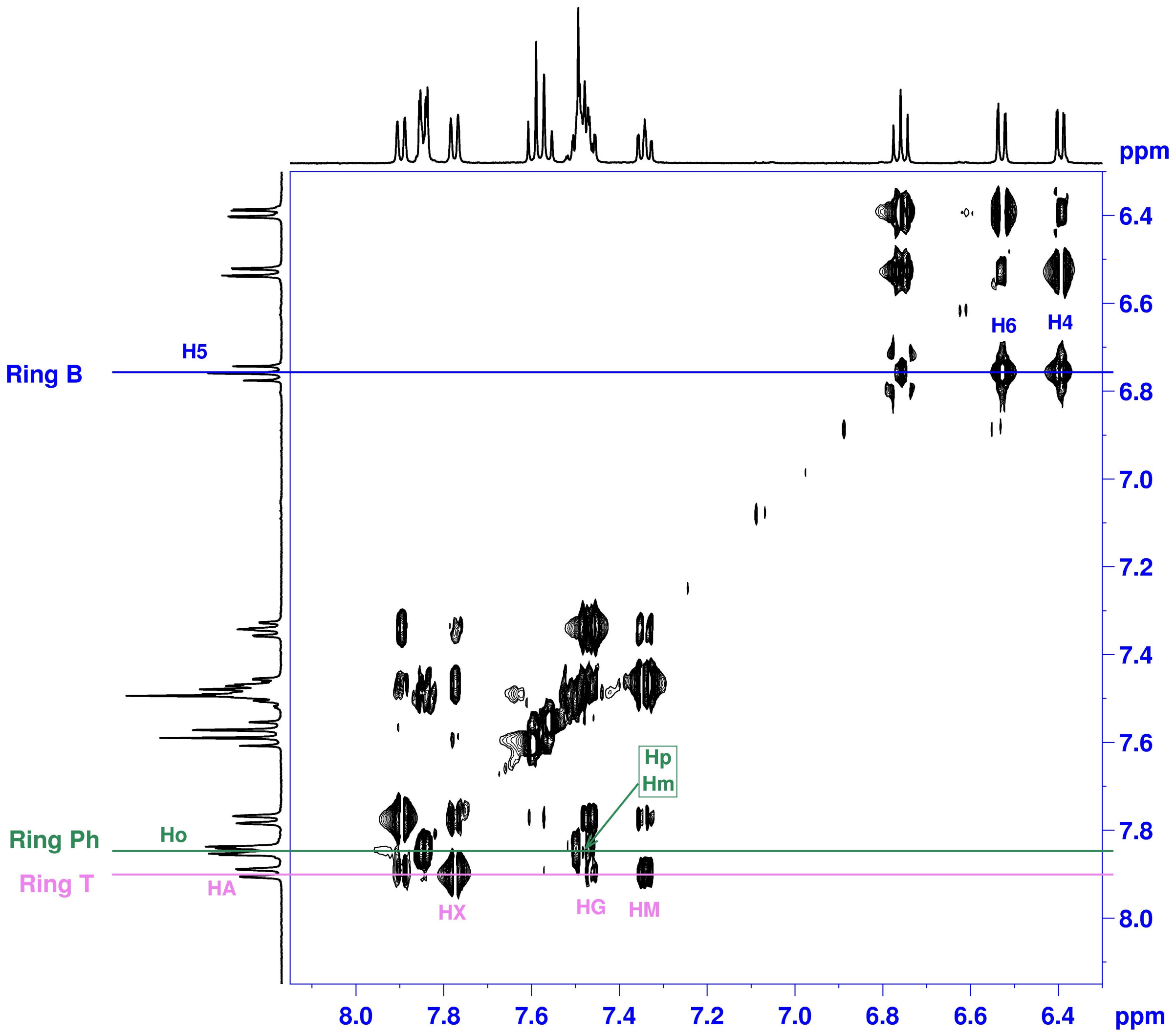

Figure S5. TOCSY $^{1}H–^{1}H$ NMR spectra of freshly generated **1[4]-H** recorded in DMSO-d_6 containing a drop of CD_2Cl_2 and D_2O at 500 MHz.

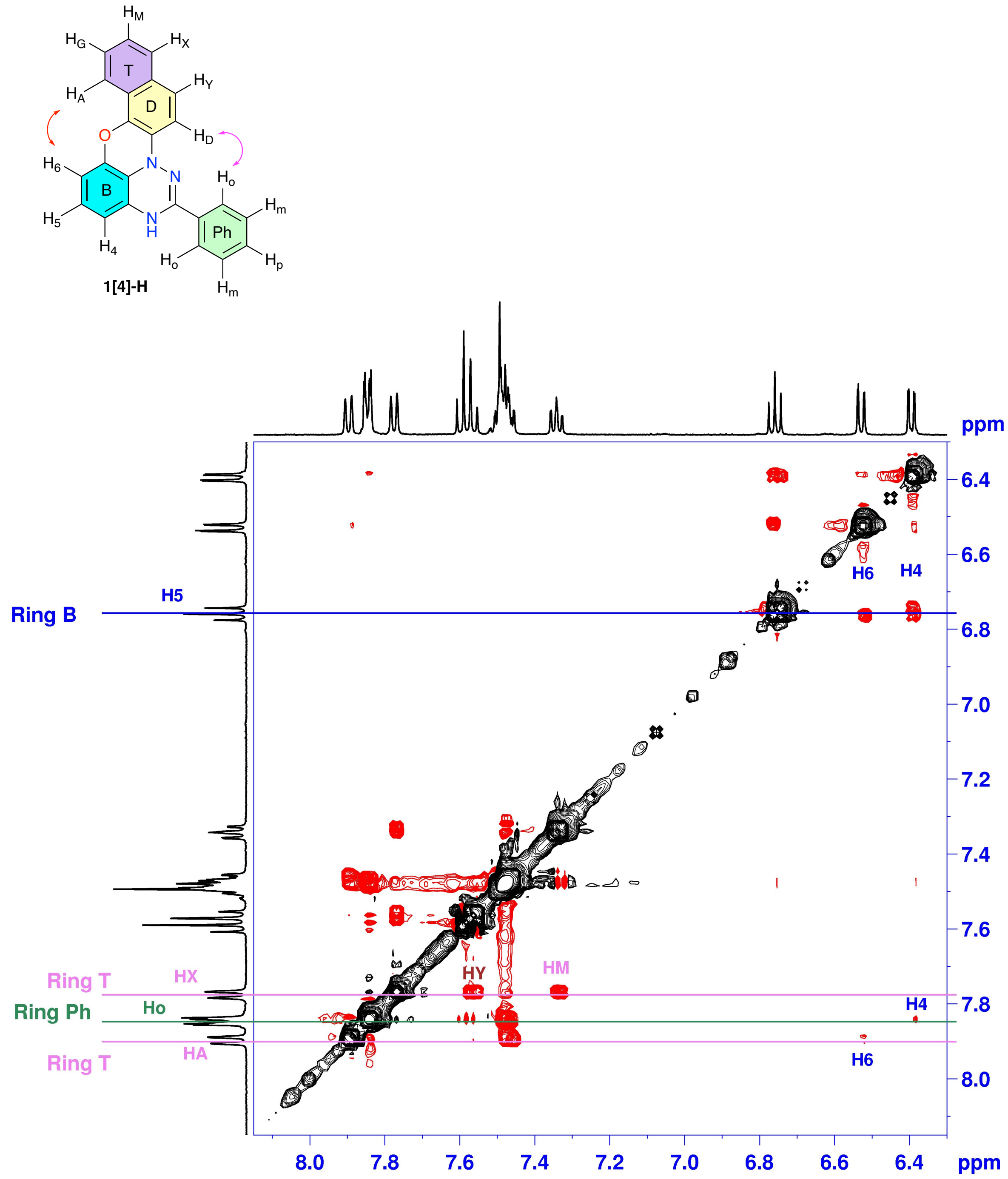

Figure S6. ROESY ^{1}H–^{1}H NMR spectra of freshly generated **1[4]-H** recorded in DMSO-d_6 containing a drop of CD_2Cl_2 and D_2O at 500 MHz.

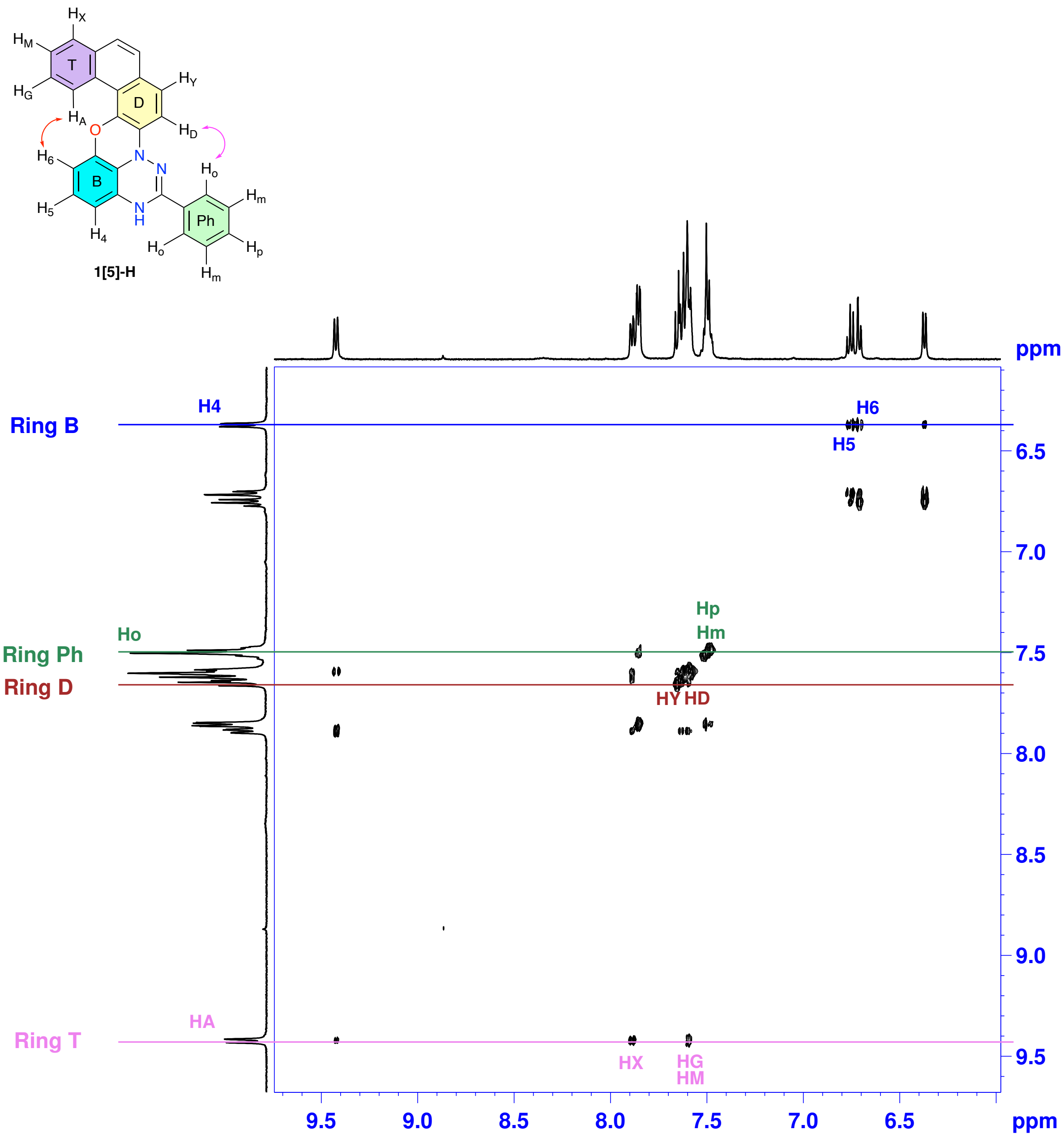

Figure S7. TOCSY ^{1}H–^{1}H NMR spectrum of freshly generated **1[5]-H** recorded in DMSO-d_6 containing a drop of CD_2Cl_2 and D_2O at 500 MHz.

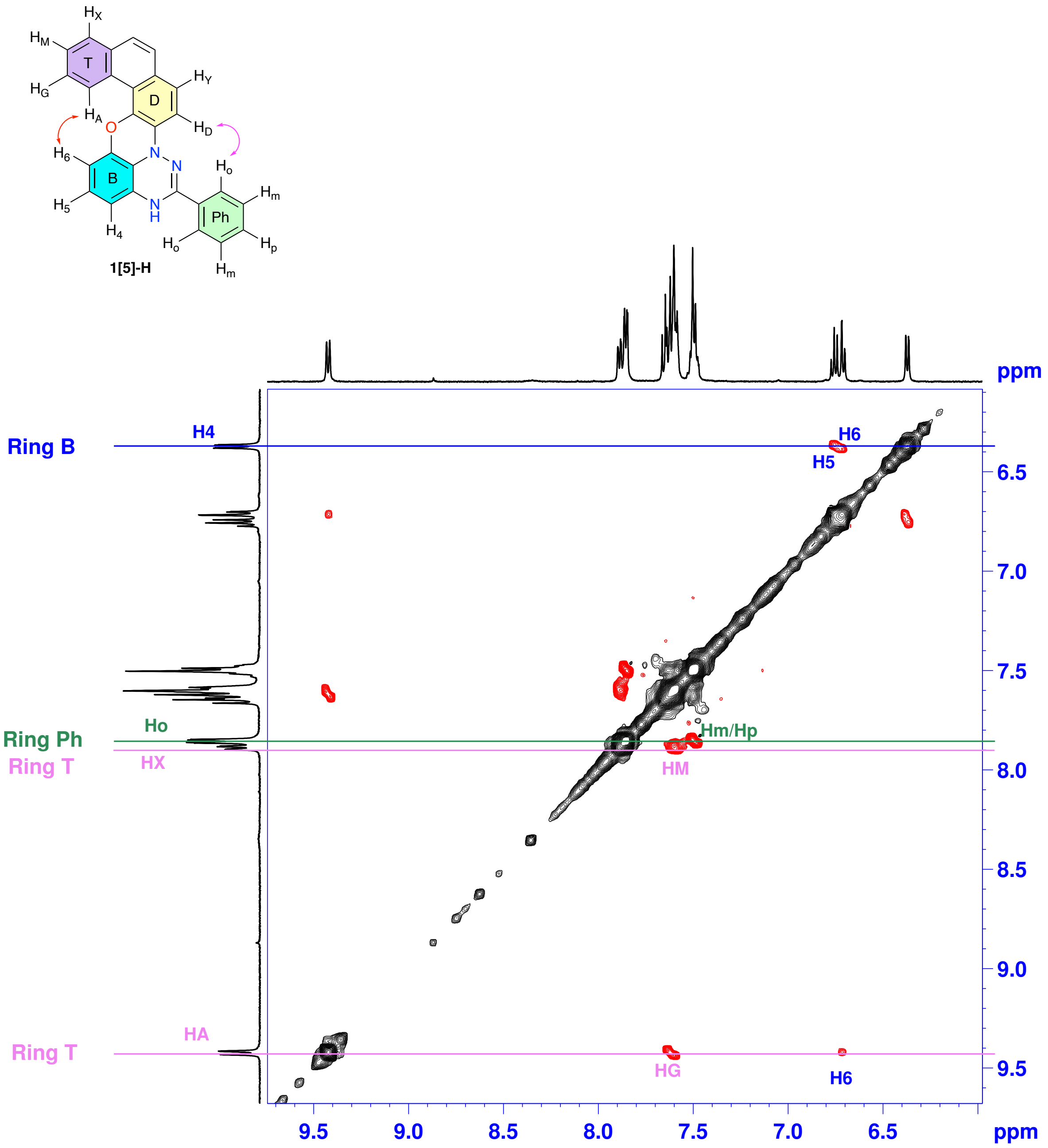

Figure S8. ROESY ^{1}H–^{1}H NMR spectrum of freshly generated **1[5]-H** recorded in DMSO-d_6 containing a drop of CD_2Cl_2 and D_2O at 500 MHz.

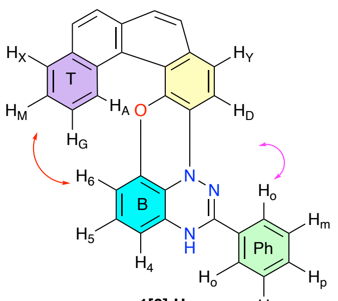

1[6]-H

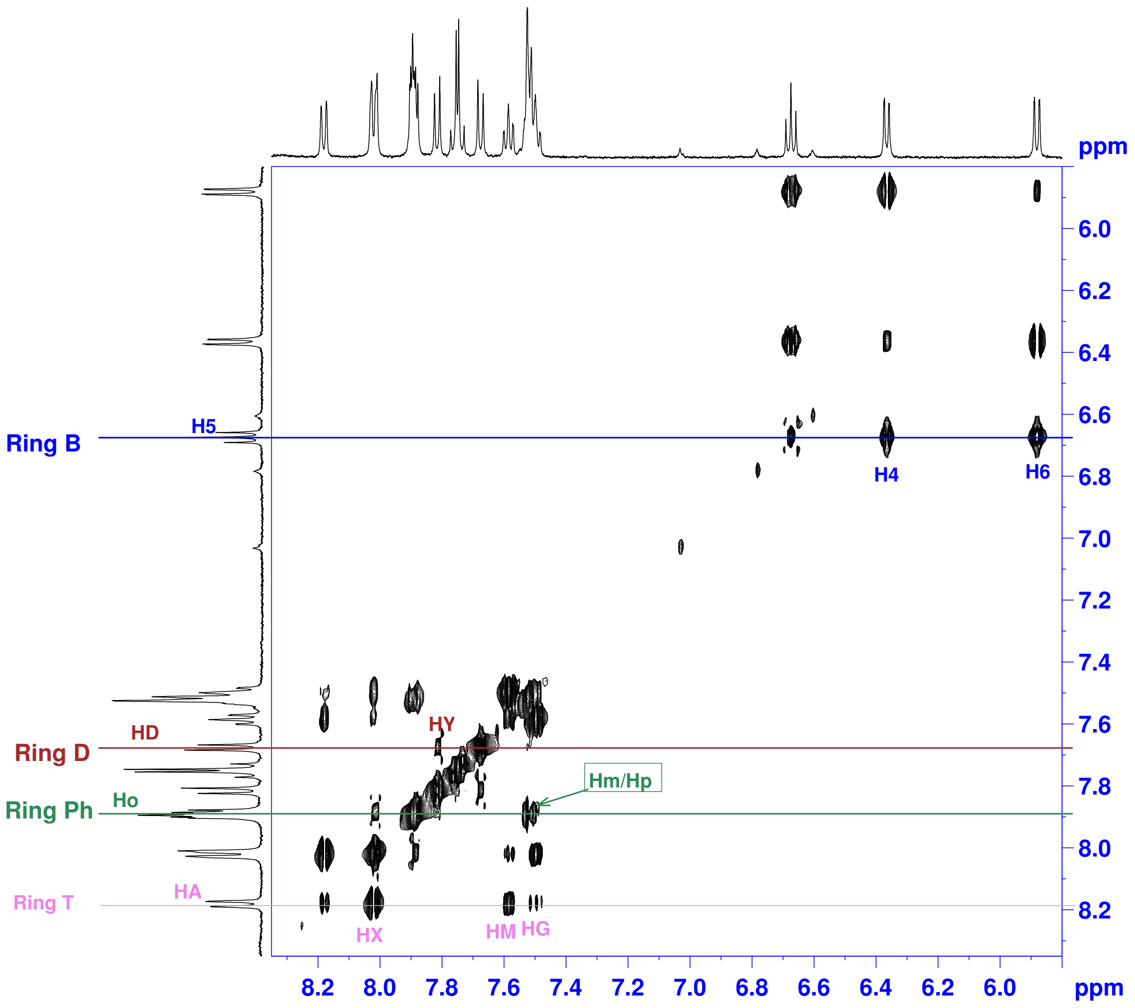

Figure S9. TOCSY ^{1}H–^{1}H NMR spectrum of freshly generated **1[6]-H** recorded in DMSO-d_6 containing a drop of CD_2Cl_2 and D_2O at 500 MHz.

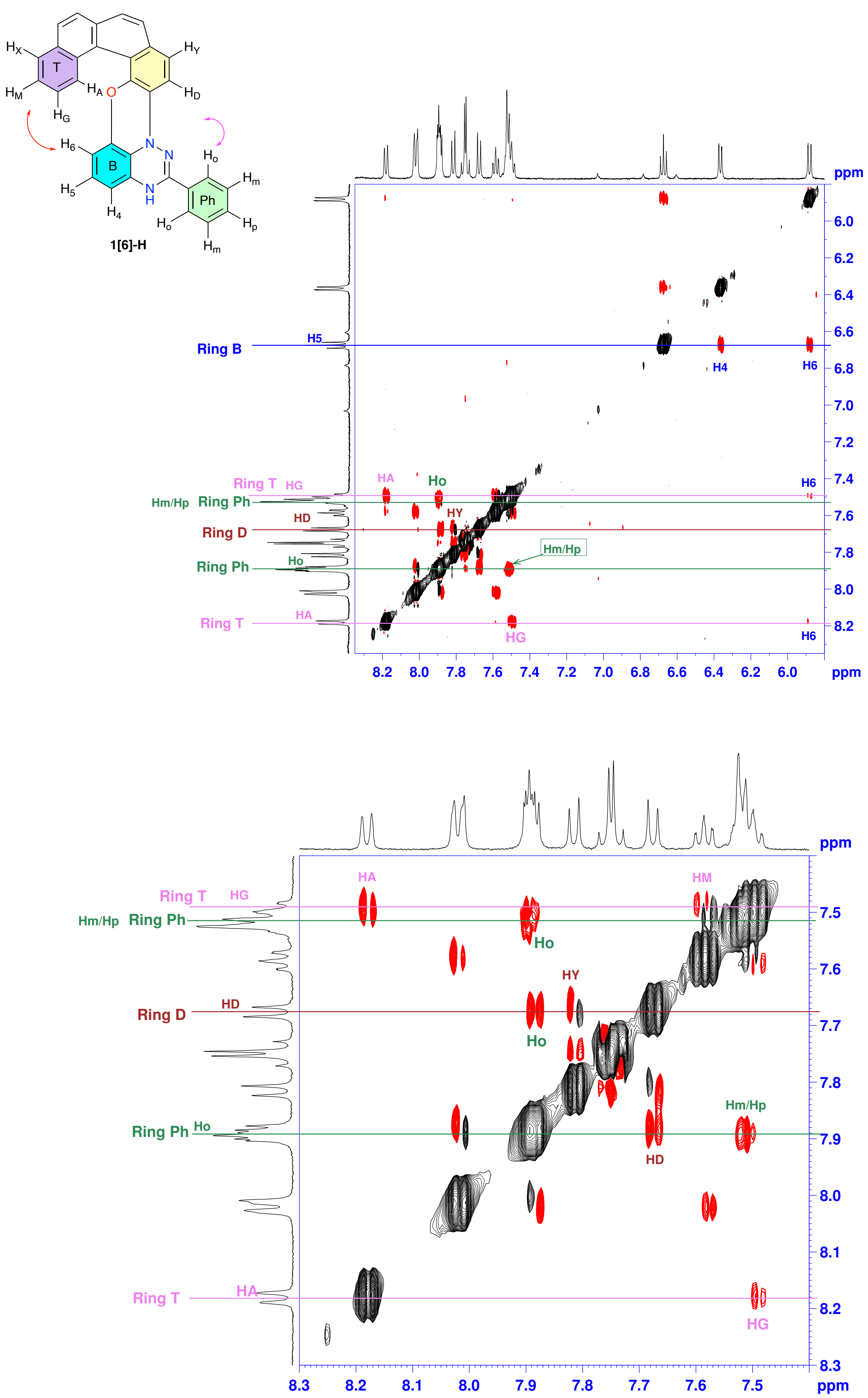

Figure S10. Two views of ROESY $^{1}H–^{1}H$ NMR spectrum of freshly generated **1[6]-H** recorded in DMSO-d_6 containing a drop of CD_2Cl_2 and D_2O at 500 MHz.

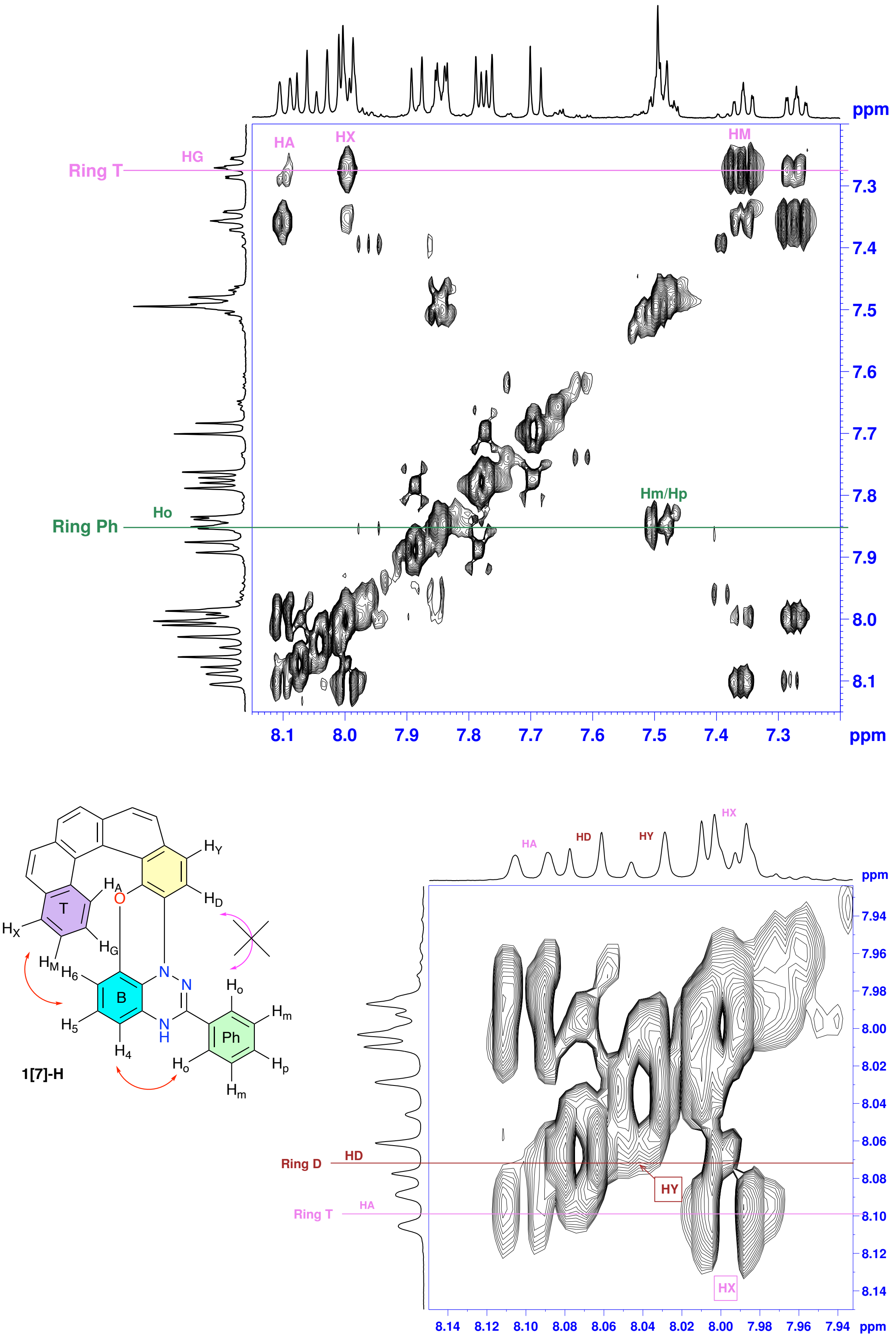

Figure S11. Two views of TOCSY ^{1}H–^{1}H NMR spectrum of freshly generated **1[7]-H** recorded in DMSO-d_6 containing a drop of CD_2Cl_2 and D_2O at 500 MHz

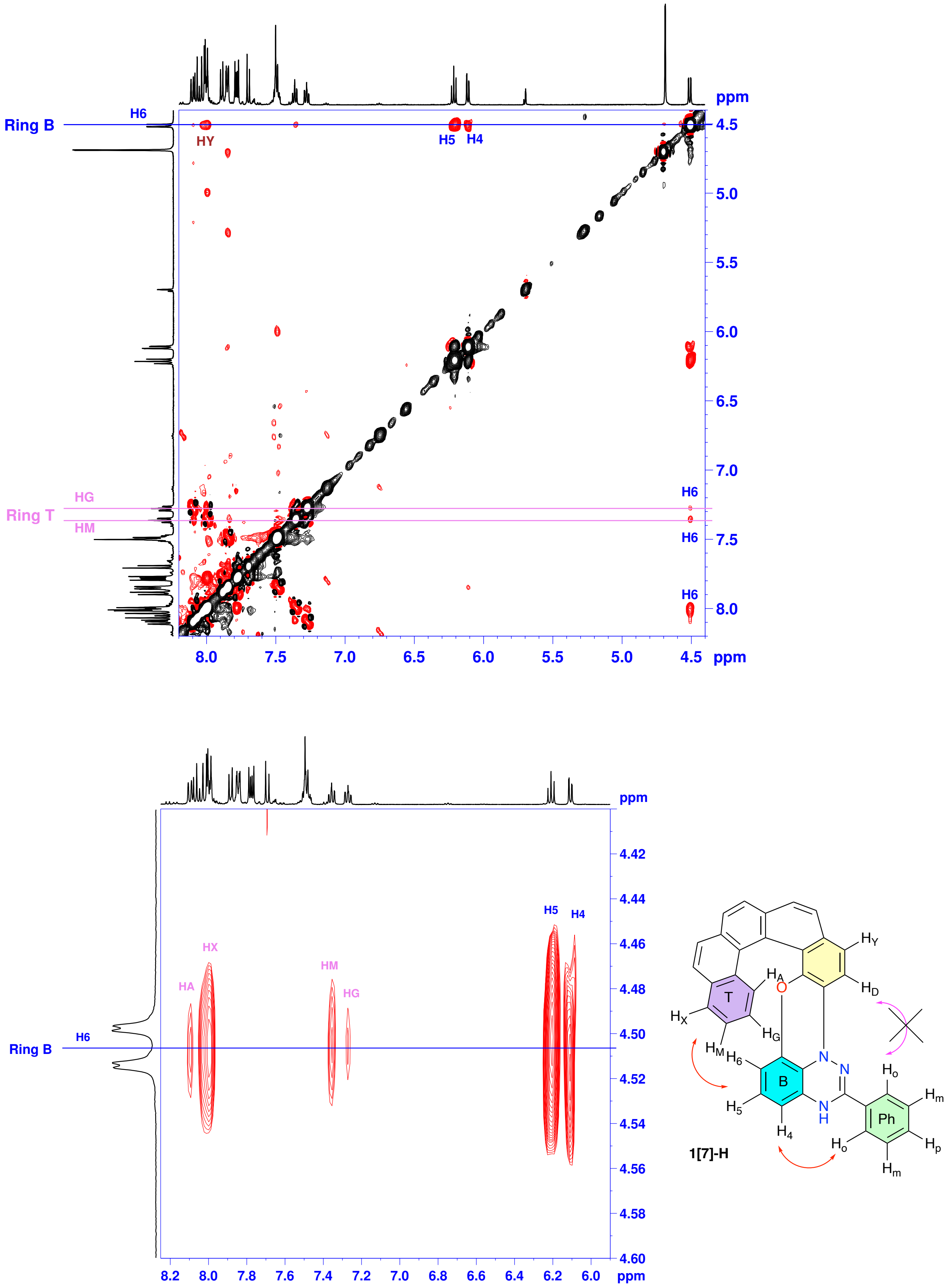

Figure S12. ROESY ^{1}H–^{1}H NMR spectrum of freshly generated **1[7]-H** recorded in DMSO-*d*$_6$ containing a drop of CD_2Cl_2 and D_2O at 500 MHz.

Table S1. Structural assignment of key ^{1}H NMR signals.

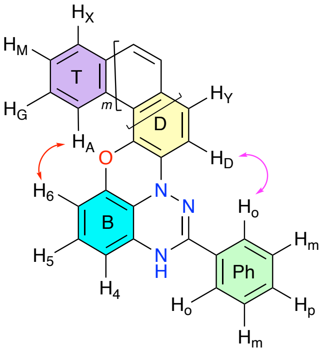

1[n]-H

leuco	H_4	H_5	H_6	H_A	H_G	H_M	H_X	H_D	H_Y	H_o	H_m	H_p
**1[4]-H**	6.39	6.76	6.53	7.89	7.46	7.35	7.77	7.59	7.52	7.85	7.49	7.49
**1[5]-H**	6.37	6.71	6.75	9.42	7.61	7.61†	7.88	7.64	7.64	7.85	7.48	7.48
**1[6]-H**	6.36	6.67	5.87	8.18	7.49	7.59	8.01	7.67	7.81	7.88	7.49	7.49
**1[7]-H**	6.11	6.22	4.51	8.10	7.27	7.36	7.99	8.01	8.00	7.84	7.49	7.49

4. Computational details

a) geometry optimization of leuco foms 1[n]-H

Quantum-mechanical calculations were carried out using Gaussian 16 suite of programs.[3] Geometry optimizations of the *leuco* forms **1[n]-H** were conducted at the B3LYP/6-31G(2d,p) level of theory in DMSO dielectric medium (PCM model[4]) requested with the SCRF(Solvent=DiMethylSulfoxide) keyword and using tight convergence limits. Fully optimized structures of *leuco* forms **1[n]-H** with indicated closed distances relevant to intramolecular through space interactions are shown in Figure S13.

b) ^{1}H NMR chemical shift calculations for 1[n]-H

GIAO isotropic magnetic shielding tensors of **1[n]-H** were calculated at the B3LYP/6-311G(2d,p) // B3LYP/6-31G(2d,p) level of theory using the NMR keyword in DMSO dielectric medium requested with the SCRF(Solvent=DiMethylSulfoxide) keyword. Chemical shifts were obtained by comparison of shielding tensors of **2[n]-H** and benzene, for which chemical shift in DMSO was assumed δ = 7.32 ppm in DMSO.[5] ^{1}H NMR chemical shift assignment is shown in Figure S14, while a comparison of

experimental chemical shifts, assigned on the basis of correlation spectroscopy, and DFT-derived shifts in shown in Figure S15.

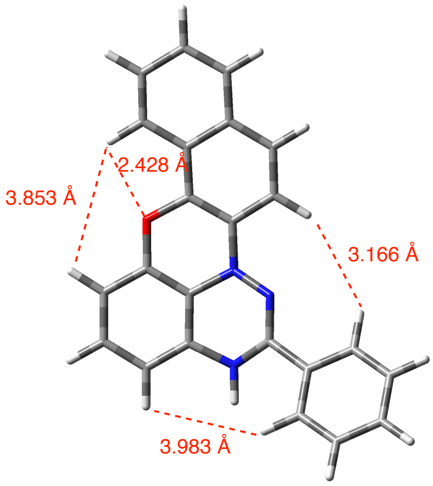

1[4]-H

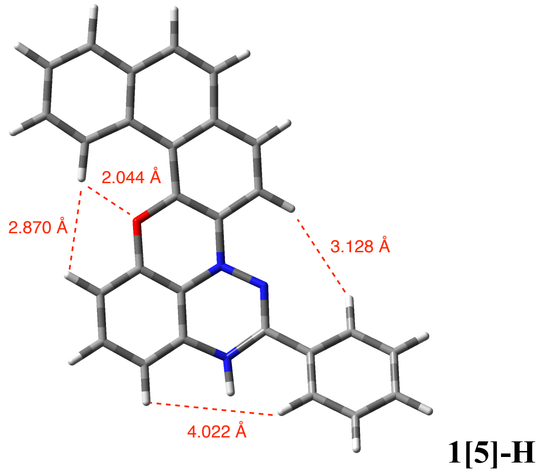

1[5]-H

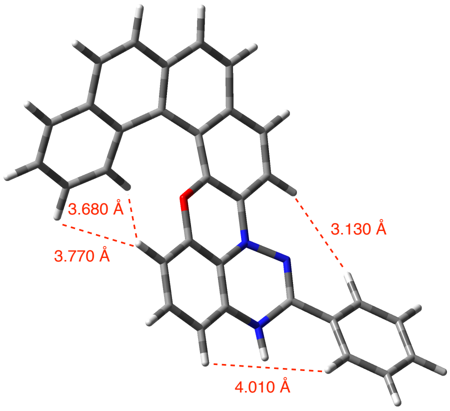

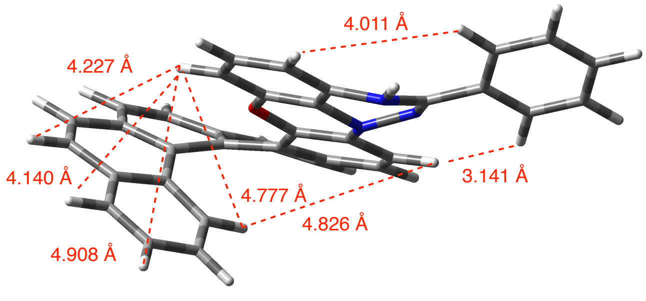

1[6]-H **1[7]-H**

Figure S13. B3LYP/6-31G(2d,p) optimized structures with key intramolecular close contacts.

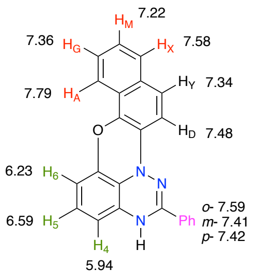

1[4]-H

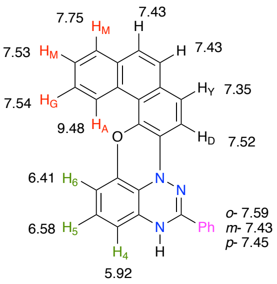

1[5]-H

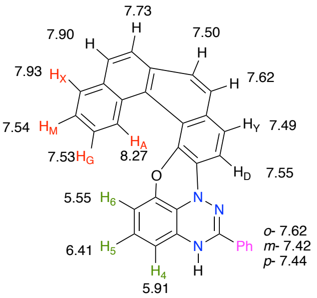

1[6]-H

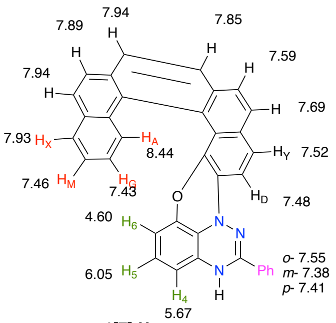

1[7]-H

Figure S14. DFT-derived ^{1}H NMR chemical shifts for *leuco* derivatives **1[n]-H**.

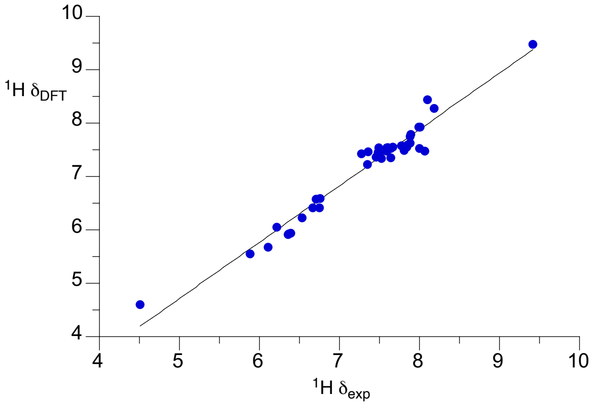

Figure S15. Experimental *vs* DFT calculated ^{1}H NMR chemical shifts for *leuco* derivatives **1[n]-H**. Best fit line: $\delta_{DFT} = 1.05(3) \times \delta_{DFT} - 0.57(25)$, $r^2 = 0.955$.

c) mechanistic investigation of photocyclization of 2[4]

Mechanistic investigation of photocyclization of **2[4]** was conducted at the (U)CAM-B3LYP/6-311G(d,p) level of theory in AcOEt dielectric medium (PCM model[4]) requested with the SCRF(Solvent=EthylEthanoate) keyword and tight convergence limits. Excitation calculations of **2[4]** were conducted using the TD-DFT method for closed-shell systems. Geometry optimization in the S_1 state was performed using Fopt and TD=(singlets, root=1, NStates=3) keywords in AcOEt dielectric medium (PCM model[4]) giving **2[4]^1**.

The triplet state geometry was obtained by optimization of the GS structure of **2[4]** in the triplet state using the UCAM-B3LYP/6-311G(d,p) method giving **2[4]^3**. TD-DFT calculations for closed-shell singlet at the triplet geometry using CAM-B3LYP/6-311G(d,p) method and TD=(triplets, root=1, NStates=12) keyword gave the forbidden S_0–>T_1 transition.

d) partial output from TD-DFT calculation for 1[4]

```
CAM-B3LYP/6-311G(d,p)// CAM-B3LYP/6-311G(d,p) in AcOEt


1[4]
Excited State 1:  Singlet-A  2.8106 eV 441.13 nm f=0.0038  <S**2>=0.000
      88 -> 92         0.65853
      88 -> 98        -0.11352
      90 -> 92         0.10154
      91 -> 92         0.11443
 This state for optimization and/or second-order correction.
 Total Energy, E(TD-HF/TD-DFT) =  -1124.50048661
 Copying the excited state density for this state as the 1-particle RhoCI
density.

Excited State 2: Singlet-A 3.7632 eV  329.47 nm  f=0.0156  <S**2>=0.000
```

```
      86 -> 92         0.13519
      88 -> 92        -0.13151
      90 -> 92         0.17287
      91 -> 92         0.63446

 Excited State 3: Singlet-A 3.9328 eV 315.26 nm  f=0.1080  <S**2>=0.000
      84 -> 92        -0.10586
      86 -> 92        -0.11683
      86 -> 93         0.11522
      89 -> 92         0.59442
      90 -> 92        -0.26564
      91 -> 92         0.12387

Excited State 4: Singlet-A 4.1348 eV  299.85 nm  f=0.0007  <S**2>=0.000
      88 -> 93         0.65311
      88 ->106         0.11373
      91 -> 93         0.12247
```

1[4]^{1} (excitation in the relaxed S_1 state geometry)

```
 Excitation energies and oscillator strengths:

 Excited State 1: Singlet-A 2.2101 eV 560.99 nm  f=0.0032  <S**2>=0.000
      90 -> 92         0.65550
      90 -> 93        -0.11845
      90 -> 98         0.13923
      91 -> 92         0.15311
 This state for optimization and/or second-order correction.
 Total Energy, E(TD-HF/TD-DFT) =  -1124.51056014
 Copying the excited state density for this state as the 1-particle RhoCI
density.

Excited State 2: Singlet-A 3.6327 eV 341.30 nm  f=0.0014  <S**2>=0.000
      90 -> 92         0.11687
      90 -> 93         0.64897
      90 ->106         0.12301
      91 -> 93         0.15170

Excited State 3: Singlet-A 3.7424 eV  331.29 nm  f=0.0200  <S**2>=0.000
      86 -> 92         0.12537
      89 -> 92         0.18126
      90 -> 92        -0.13162
      91 -> 92         0.63072
```

1[4]^{3} (excitation in the relaxed triplet state geometry)

```
Excited State 1:  Triplet-A 1.5727 eV 788.36 nm  f=0.0000  <S**2>=2.000
      89 -> 92        -0.11385
      90 -> 92         0.62711
      90 -> 93        -0.17188
      90 -> 98         0.17745
      91 -> 92         0.11088
 This state for optimization and/or second-order correction.
 Total Energy, E(TD-HF/TD-DFT) =  -1124.53709136
 Copying the excited state density for this state as the 1-particle RhoCI
density.
```

```
Excited State 2: Triplet-A 2.3754 eV  521.95 nm  f=0.0000  <S**2>=2.000
      86 -> 92         -0.28385
      86 -> 93          0.14466
      88 -> 92          0.52058
      88 -> 93          0.11981
      89 -> 92         -0.21011
      88 <- 92          0.10161

Excited State 3: Triplet-A 2.7071 eV  457.99 nm  f=0.0000  <S**2>=2.000
      85 -> 99          0.17586
      89 -> 94         -0.18856
      89 -> 95          0.16323
      90 -> 94         -0.11246
      91 -> 94          0.59321
      91 -> 95          0.12598
      91 <- 94          0.12329

Excited State 4: Triplet-A 3.2184 eV  385.24 nm  f=0.0000  <S**2>=2.000
      84 -> 93          0.13679
      86 -> 92          0.44676
      86 -> 98         -0.11788
      87 -> 92         -0.10525
      87 -> 96          0.19994
      88 -> 92          0.20573
      88 -> 93          0.24103
      88 -> 95          0.11055
      88 -> 97         -0.16033
      91 -> 92          0.16197
```

Geometries of transition states in AcOEt dielectric medium were located using the QST3 algorithm implemented with the Opt(QST3, CalcFC) keyword and restricted CAM-B3LYP/6-311G(d,p) method with default convergence limits. The input geometry for the TS was obtained from PES relaxed scans.

e) N–H bond dissociation energy

The homolytic bond dissociation enthalpy (*HBDE*) for tautomeric species **6[4]**, **1[4]-1H** and **1[4]-H** was calculated at the (U)CAM-B3LYP/6-311G(d,p) level of theory in AcOEt dielectric medium (requested with the SCRF(Solvent=EthylEthanoate) keyword; PCM model[4])) as a change of enthalpy ΔH in an isodesmic reaction with the structurally similar phenoxazin-10-yl radical (Scheme S1) and referenced to the experimental C–H *HBDE* of phenoxazine in benzene (77.2±0.3 kcal mol^{-1}).[6] Energies or relevant molecules are collected in Table S2.

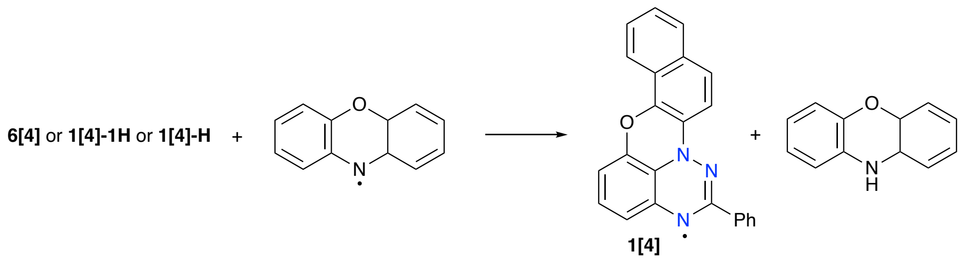

Scheme S1. Isodesmic reaction to calculate HBDE in tautomeric *leuco* forms of radical **1[4]**.

Table S2. DFT calculated energies and thermodynamic corrections for photocyclization of **2[4]** in AcOEt dielectric medium. [a]

species	E_{SCF} /Ha	ZPEC /Ha	H corr /Ha	G_{298} corr /Ha
**2[4]**	-1124.60377448	0.32639	0.346672	0.275492
**2[4]**^{1} [b]	-1124.50048661			
**2[4]**^{1} [c]	-1124.51056014			
**2[4]**^{3} [d]	-1124.53383273	0.323681	0.344509	0.270903
**4[4]**^{3} [d]	-1124.53640464	0.324508	0.34456	0.27418
**5[4]**	1124.58454575	0.326218	0.346648	0.276059
**TS-1'**	-1124.559127	0.325885	0.345306	0.278262
**6[4]**	-1124.58875295	0.327669	0.347236	0.280009
**1[4]-1H**	-1124.60666757	0.328244	0.34794	0.280379
**1[4]-H**	-1124.62030885	0.328351	0.348128	0.280683
**1[4]** [e]	-1124.00915496	0.316001	0.335445	0.266494
**TS-3'**	-1124.54020200	0.325549	0.344926	0.277991
**7[4]**	-1124.55491220	0.327669	0.347236	0.280009
**phenoxazine**	-591.8797079	0.16928	0.179528	0.133782
**phenoxazinyl**[e]	-592.5064784	0.181992	0.192669	0.14657

[a] Obtained at the CAM-B3LYP/6-311G(d,p) level of theory. [b] SCF energy of the S_1 state obtained at the TD-CAM-B3LYP/6-311G(d,p)// CAM-B3LYP/6-311G(d,p) level of theory. [c] SCF energy of the relaxed S1 state obtained at the TD-CAM-B3LYP/6-311G(d,p) level of theory. [d] Triplet state optimized at the UCAM-B3LYP/6-311G(d,p) level of theory. [e] Doublet state optimized at the UCAM-B3LYP/6-311G(d,p) level of theory.

5. Archive for DFT calculations

1[4]

1\1\GINC-LOCALHOST\FOpt\UB3LYP\6-311G(d,p)\C23H14N3O1(2)\PIOTR\27-Jul-
2024\0\\#P UB3LYP/6-311G(d,p) FOpt=tight freq(noraman) SCF=Direct #P G
eom=(NoDistance,NoAngle) fcheck SCRF(Solvent=CH2Cl2)\\benzotrazinyl 1,
2-naphthyl, Cs, Smiles\\0,2\N,0.1360787742,-0.1732928935,0.\N,1.470078
6957,-2.579376815,0.\C,0.1407303001,-2.4582983309,0.\N,-0.5749400497,-
1.3247903862,0.\C,3.6195139616,1.026907499,0.\C,2.2346733602,1.0364571
264,0.\C,1.527472918,-0.1669211033,0.\C,2.1901588663,-1.4126311102,0.\
C,3.596574715,-1.406536373,0.\C,4.2886020228,-0.2044366529,0.\C,-0.544
7192359,1.0538984021,0.\C,0.1953693831,2.2294397141,0.\C,-0.4411620144
,3.4963909334,0.\C,-1.8711663155,3.5394103616,0.\C,-1.9607180393,1.110
402639,0.\C,-0.6643740691,-3.7124427496,0.\C,-2.0662901119,-3.67998793
34,0.\C,-2.7993780121,-4.8625573522,0.\C,-2.1461798709,-6.0951623121,0
.\C,-0.752339903,-6.1361747225,0.\C,-0.0163806121,-4.9550600471,0.\O,1
.5720654166,2.2416815271,0.\H,4.113728359,-2.3574324306,0.\H,-2.507161
1261,0.1791790193,0.\H,-2.5741074111,-2.7250273833,0.\H,-3.8828284371,
-4.8223342211,0.\H,-0.2369127296,-7.0900143053,0.\H,1.0649085526,-4.97
72109182,0.\H,5.3719138371,-0.2104331548,0.\H,4.1575727056,1.965869564
6,0.\H,-2.7190897672,-7.0156090553,0.\C,0.2925022529,4.7118294854,0.\C
,-0.3667795771,5.9184122106,0.\C,-2.5124550821,4.8027136952,0.\C,-1.77
97186384,5.9669777393,0.\C,-2.6009754505,2.3199954643,0.\H,0.200492086
6,6.8419337252,0.\H,-2.2835851561,6.926481403,0.\H,1.3736184112,4.6752
417587,0.\H,-3.5964857216,4.8350803838,0.\H,-3.6841187879,2.3567933784
,0.\\Version=ES64L-G16RevC.01\State=2-A"\HF=-1124.6058951\S2=0.765927\
S2-1=0.\S2A=0.750207\RMSD=7.163e-09\RMSF=1.315e-06\Dipole=-0.1991871,1
.4390989,0.\Quadrupole=9.1165202,6.0167656,-15.1332858,1.1490935,0.,0.
\PG=CS [SG(C23H14N3O1)]\\

1[4]-H

1\1\GINC-LOCALHOST\FOpt\RB3LYP\6-31G(2d,p)\C23H15N3O1\PIOTR\04-Dec-202
3\0\\#P B3LYP/6-31G(2d,p) FOpt(tight) SCF=Direct #P Geom=(NoDistance,N
oAngle) fcheck SCRF(Solvent=DiMethylSulfoxide)\\4-Ph OxoNaphthalene he
licene leuco form isomer\\0,1\N,-0.9830885438,-0.0051687967,-0.6047928
133\N,-3.2120934556,1.4479960277,-0.6095175571\C,-3.2401074342,0.09907
93368,-0.2584162959\N,-2.178643712,-0.6326924069,-0.2110101677\C,0.454
555727,3.3596413062,0.0872655616\C,0.3659544976,1.9831806302,-0.115160
4907\C,-0.8624988937,1.3847614702,-0.3517715735\C,-2.0217382406,2.1573
617319,-0.3646337012\C,-1.954918389,3.5298739027,-0.1459586814\C,-0.70
84128012,4.1244613247,0.0688265331\C,0.1898599329,-0.7648681898,-0.427
1788701\C,1.4039481024,-0.1315694891,-0.2123449012\C,2.6097601015,-0.8
638524139,-0.0852931086\C,0.1570628041,-2.1779275202,-0.5127917349\C,2
.5586133794,-2.2921354354,-0.1753456888\C,1.3057829562,-2.9188456586,-
0.3894518356\O,1.5260243549,1.2460272257,-0.1188200206\H,-2.8613776395
,4.1249836068,-0.1525996224\H,-0.6465717994,5.1960534075,0.2207824231\
H,1.428479457,3.8065266119,0.2479893002\C,-4.5442657254,-0.5176101389,
0.0644940512\C,-4.7568152591,-1.8774291571,-0.2087168921\C,-5.58053446
89,0.2269628921,0.6455588405\C,-5.9747157493,-2.4751678385,0.093188132
1\H,-3.9601813337,-2.4505194922,-0.6675891927\C,-6.8002398723,-0.37542
01019,0.946383796\H,-5.433356922,1.2716955702,0.8985218284\C,-7.002270
8583,-1.7261586637,0.6702463951\H,-6.1263920135,-3.5261732245,-0.12939
53247\H,-7.5893364593,0.2128510002,1.4024452523\H,-7.9538211544,-2.193
1806273,0.9011638926\H,-4.0773879024,1.9622200698,-0.5440190442\H,-0.8
00647765,-2.6539350934,-0.6754371254\H,1.262536942,-4.0010995507,-0.45
84275468\C,3.8623834177,-0.2302052089,0.1376433837\C,3.7641638985,-3.0
275727089,-0.0449089971\C,5.0094441753,-0.9784972841,0.2609446513\C,4.
9639774846,-2.3900414805,0.1679248155\H,5.8783881737,-2.965647351,0.26
62174287\H,3.7195268578,-4.1104097645,-0.1156105558\H,3.8989702429,0.8
497706153,0.2085431191\H,5.9601211371,-0.4832232133,0.4304171074\\Vers

ion=ES64L-G09RevD.01\State=1-A\HF=-1124.9981425\RMSD=9.452e-09\RMSF=8.
682e-07\Dipole=-1.7715809,0.7705276,0.2295537\Quadrupole=11.3353017,5.
4520546,-16.7873562,-4.4743242,-1.0504406,2.3267042\PG=C01 [X(C23H15N3
O1)]\\@

1[5]-H

1\1\GINC-LOCALHOST\FOpt\RB3LYP\6-31G(2d,p)\C27H17N3O1\PIOTR\02-Dec-202
3\0\\#P B3LYP/6-31G(2d,p) FOpt(tight) SCF=Direct #P Geom=(NoDistance,N
oAngle) fcheck SCRF(Solvent=DiMethylSulfoxide)\\4-Ph Oxophenanthrere h
elicene leuco form isomer\\0,1\N,-0.9799199669,0.036267365,-0.57585206
75\N,-3.2375714533,1.4630002956,-0.5865760337\C,-3.2440986469,0.111575
2866,-0.2506990027\N,-2.1732746792,-0.6070383029,-0.2043729051\C,0.418
0454451,3.4150248226,0.0538813475\C,0.3364807276,2.0354080602,-0.13274
06921\C,-0.8864932363,1.4260351648,-0.344481043\C,-2.0538420593,2.1861
443507,-0.356373198\C,-1.9963710459,3.5610907717,-0.1541534381\C,-0.75
23069404,4.1686587603,0.0411786946\C,0.1973802144,-0.7174866493,-0.408
1059894\C,1.4300834545,-0.0793768468,-0.2172906863\C,2.6466081452,-0.8
035416873,-0.0936829929\C,0.1440093154,-2.1184285917,-0.4703399155\C,2
.5459379658,-2.2330000914,-0.155296766\C,1.2973611601,-2.8539056717,-0
.3424967838\O,1.4989451052,1.3036747268,-0.1503245868\H,-2.9080862306,
4.148252565,-0.1590320314\H,-0.6983233258,5.2422512772,0.1810381462\H,
1.3879837882,3.87564032,0.1985757124\C,-4.5401722411,-0.5282424323,0.0
604291718\C,-4.7387336807,-1.8822973338,-0.2481978567\C,-5.580827981,0
.1893986392,0.666910571\C,-5.9481477301,-2.5017312573,0.0445627146\H,-
3.9385103501,-2.4334807144,-0.7272304985\C,-6.7915890401,-0.4348724358
,0.9589669302\H,-5.4428961346,1.2285228939,0.9468760456\C,-6.980041077
4,-1.7800333978,0.6479244807\H,-6.0896731488,-3.5479394517,-0.20549232
81\H,-7.5844673764,0.1316643566,1.4354922458\H,-7.9248193283,-2.263770
2197,0.8720862421\H,-4.1095088825,1.9654989728,-0.5222617376\H,-0.8163
88874,-2.5932906928,-0.6141268507\H,1.2559912621,-3.9373000558,-0.3871
964641\C,3.9789023077,-0.2122680481,0.1007468369\C,3.710723077,-3.0521
654977,-0.020342816\C,5.1035194141,-1.0892910375,0.2399354008\C,4.9362
610234,-2.5097531763,0.1750222435\H,5.8147470087,-3.1386287654,0.28136
75715\H,3.5811437152,-4.1288266999,-0.0738963604\C,6.3990650238,-0.562
044143,0.4367141165\C,4.2526900126,1.1782949701,0.1570083635\C,6.62175
71286,0.7970573829,0.4935446433\H,7.6231744977,1.186421475,0.644894913
2\C,5.5328631861,1.6677797396,0.3476820544\H,5.6879426671,2.7414579237
,0.3825728325\H,3.4493028602,1.8840733495,0.0448920609\H,7.2248679238,
-1.2596532693,0.5409487052\\Version=ES64L-G09RevD.01\State=1-A\HF=-127
8.6442846\RMSD=3.408e-09\RMSF=7.765e-07\Dipole=-2.0848563,0.8279477,0.
1980263\Quadrupole=15.1465757,5.0698588,-20.2164345,-4.7126966,-0.9243
147,2.0979135\PG=C01 [X(C27H17N3O1)]\\@

1[6]-H

1\1\GINC-LOCALHOST\FOpt\RB3LYP\6-31G(2d,p)\C31H19N3O1\PIOTR\02-Dec-202
3\0\\#P B3LYP/6-31G(2d,p) FOpt(tight) SCF=Direct #P Geom=(NoDistance,N
oAngle) fcheck SCRF(Solvent=DiMethylSulfoxide)\\4-Ph OxoBenzphenanthre
ne helicene leuco form isomer\\0,1\N,-1.0016983653,0.0947446204,-0.352
8589739\N,-3.2566860016,1.5078132551,-0.324746952\C,-3.2764260486,0.12
26183618,-0.1736064744\N,-2.2041572757,-0.5969764624,-0.1549461574\C,0
.3251005439,3.3439969984,0.8564677765\C,0.2705327741,2.00495407,0.4714
840346\C,-0.9287322114,1.4387829195,0.0741161969\C,-2.0986745225,2.193
67281,0.0849685676\C,-2.0688588248,3.5250934937,0.4878085514\C,-0.8480
120622,4.0941587452,0.8625446869\C,0.1803248833,-0.6557230725,-0.31133
61023\C,1.3932635001,-0.0320595811,-0.000385926\C,2.6274529762,-0.7281
086091,-0.0541617562\C,0.1649406026,-2.0345435397,-0.5941339509\C,2.55
18210546,-2.1560261458,-0.1740326864\C,1.3208082536,-2.7704960897,-0.4
812222072\O,1.429613029,1.270670768,0.4762658336\H,-2.9825796065,4.108
8716321,0.5013031336\H,-0.8141923817,5.1361131281,1.1598753954\H,1.278
3826196,3.773379746,1.139506283\C,-4.5853225333,-0.5512261607,-0.04146
19843\C,-4.7445307102,-1.8599351144,-0.521998946\C,-5.6796050701,0.088

47903,0.5580401306\C,-5.9669280831,-2.5105740202,-0.4039149153\H,-3.90
27208711,-2.3503504223,-0.9956891568\C,-6.9035515237,-0.566881868,0.67
44547242\H,-5.5773225525,1.0876001829,0.9685214279\C,-7.0522053541,-1.
8660597839,0.1932786303\H,-6.076424907,-3.5201678483,-0.7858184348\H,-
7.738314624,-0.0607650165,1.1474527682\H,-8.0069474936,-2.3736480234,0
.2808083392\H,-4.135441223,1.9964210157,-0.2477274543\H,-0.7812400885,
-2.5021414891,-0.8294420762\H,1.2912997145,-3.8483293298,-0.6039307721
\C,3.9415714022,-0.1184489165,0.1230932437\C,3.6976752343,-2.950465652
3,0.1238694891\C,4.9922381098,-0.9518235995,0.5792523449\C,4.842444748
6,-2.3731650611,0.5803937946\H,5.6859600439,-2.9805263207,0.8933188719
\H,3.6088038635,-4.0298715053,0.0481029236\C,6.2338722356,-0.379008628
2,0.9946361429\C,4.2773820068,1.2510909798,-0.2188110926\C,6.467695162
1,0.9573178266,0.8841146651\H,7.4026391547,1.3895369568,1.2276578582\C
,5.5258217183,1.7968453026,0.2182951443\H,6.9885113078,-1.040644537,1.
4091679045\C,5.8532974074,3.1401745792,-0.0851849302\C,3.4895474305,2.
0544101182,-1.0827431354\C,3.8524429631,3.3457483397,-1.4023951043\H,3
.2324367901,3.9241910809,-2.0797351531\C,5.0270026105,3.9129146286,-0.
8701450813\H,5.2953736635,4.9365951475,-1.1096452661\H,6.792790538,3.5
385317872,0.286506878\H,2.6069496022,1.6303447235,-1.5406893713\\Versi
on=ES64L-G09RevD.01\State=1-A\HF=-1432.2817686\RMSD=6.947e-09\RMSF=8.6
41e-07\Dipole=-2.0536093,0.6685618,0.1221533\Quadrupole=18.2650492,2.3
761905,-20.6412397,-3.6751876,1.5201736,1.960901\PG=C01 [X(C31H19N3O1)
]\\@

1[7]-H

1\1\GINC-LOCALHOST\FOpt\RB3LYP\6-31G(2d,p)\C35H21N3O1\PIOTR\03-Dec-202
3\0\\#P B3LYP/6-31G(2d,p) FOpt(tight) SCF=Direct #P Geom=(NoDistance,N
oAngle) fcheck SCRF(Solvent=DiMethylSulfoxide)\\4-Ph OxoNaphthphenanth
rene helicene leuco form isomer\\0,1\N,-1.5746706016,-0.1274638602,-0.
4042015278\N,-3.6111114907,1.5831579517,-0.3130779333\C,-3.809722917,0
.2202056443,-0.1009675421\N,-2.8462939083,-0.6395477144,-0.1100914444\
C,0.2517525852,2.9486419419,0.5870828371\C,-0.0078735785,1.6162472648,
0.268263802\C,-1.2947489195,1.2069489241,-0.0364436187\C,-2.3481931401
,2.1167426488,0.0033210478\C,-2.1132463121,3.4454611397,0.3421009214\C
,-0.8055625946,3.8539451671,0.6220247188\C,-0.505524176,-1.0342991316,
-0.3800410164\C,0.7932900012,-0.5781968653,-0.1356872024\C,1.914992905
8,-1.4410749809,-0.202602639\C,-0.7226389794,-2.4028531357,-0.62425306
45\C,1.6395854036,-2.845267785,-0.2922617777\C,0.3259327529,-3.2889560
287,-0.545671299\O,1.0393424068,0.7302135938,0.24119521\H,-2.936595321
5,4.1502666476,0.3782528258\H,-0.6121878187,4.8919857806,0.8676491177\
H,1.2703250449,3.2519337956,0.7962871207\C,-5.1874758672,-0.2607972896
,0.1340885232\C,-5.5553208091,-1.5484147378,-0.2849058196\C,-6.1423374
323,0.5431031924,0.7729202751\C,-6.8454083768,-2.0181486083,-0.0687624
357\H,-4.8208406446,-2.1656853527,-0.7879919918\C,-7.4344478242,0.0686
680602,0.9882229598\H,-5.8767646343,1.5302636354,1.136608666\C,-7.7908
81468,-1.2108564197,0.5671285667\H,-7.1172421736,-3.0135487427,-0.4040
75628\H,-8.1594495056,0.7001079447,1.4905754012\H,-8.7986464903,-1.577
484016,0.7310221203\H,-4.4095823625,2.1904329782,-0.2106107671\H,-1.73
31266237,-2.7391949481,-0.8118757766\H,0.144862056,-4.3541365583,-0.64
67381781\C,3.3024995264,-1.0060164117,-0.0346946147\C,2.6720921845,-3.
7873691319,-0.0068132597\C,4.2102350693,-1.9739526549,0.4807503034\C,3
.8778255523,-3.3648542038,0.4581332791\H,4.6290872258,-4.0761368992,0.
786688841\H,2.4403668567,-4.8453598353,-0.0808189672\C,5.4635377688,-1
.5659278306,1.0130215975\C,3.8306654151,0.3095246585,-0.3302202018\C,5
.8143207381,-0.2474787101,1.0254471901\H,6.7327826292,0.0813002453,1.5
017800357\C,5.0307583827,0.7065699772,0.3219493343\H,6.1120466877,-2.3
204568162,1.4473056158\C,5.4862545995,2.0600242601,0.237840878\C,3.296
0309459,1.2189574288,-1.3361845843\C,3.7832013073,2.5603761067,-1.4014
163171\C,4.8512191525,2.9713982884,-0.545314392\H,5.1921584297,4.00114
80195,-0.5924993855\H,6.3604918087,2.3402497217,0.8177805\C,2.39015481
14,0.812212241,-2.346159596\C,3.2606876491,3.4543202995,-2.3664592681\

C,1.9208247574,1.6916508192,-3.3000134905\H,1.2340104279,1.3387404623,
-4.0625929797\C,2.3355036751,3.0368195216,-3.2973411582\H,2.0700335951
,-0.2200858713,-2.3864519688\H,3.6311461043,4.475227954,-2.3770658134\
H,1.9524593831,3.7283554857,-4.0405352693\\Version=ES64L-G09RevD.01\St
ate=1-A\HF=-1585.9245294\RMSD=5.297e-09\RMSF=1.547e-06\Dipole=-1.82657
2,0.9274415,0.2560124\Quadrupole=16.5185608,3.5015872,-20.0201481,-4.4
656424,2.2001594,1.7776081\PG=C01 [X(C35H21N3O1)]\\@

Mechanistic studies

2[4]

1\1\GINC-LOCALHOST\FOpt\RCAM-B3LYP\6-311G(d,p)\C23H15N3O1\PIOTR\01-Aug
-2024\0\\#P CAM-B3LYP/6-311G(d,p) FOpt=tight SCF=Direct freq(noraman)
#P Geom=(NoDistance,NoAngle) fcheck SCRF(Solvent=EthylEthanoate)\\3Ph-b
enzotrazinyl naphth-2-oxy opt in ground state in vacuumm\\0,1\N,1.6512
1263,0.9130917062,-1.335302603\N,3.8214484958,1.793195038,0.1061272879
\C,3.2319629603,2.4985507843,-0.8292160649\N,2.1566087719,2.0741588265
,-1.5608225348\C,2.2207973174,-1.9560992722,0.8240451144\C,1.646539166
8,-1.1590020436,-0.1190092377\C,2.1943037159,0.1297981044,-0.377843457
4\C,3.3144359942,0.5714540508,0.3581499805\C,3.8915904433,-0.271344637
9,1.336844781\C,3.3508681147,-1.5050777051,1.5505630857\C,-1.041011126
1,-0.4229911509,0.4315325779\C,-0.662063767,-1.1248500123,-0.674977820
3\C,-1.5827294262,-1.4324633731,-1.7001600715\C,-2.3821406691,0.020729
6254,0.5580030215\C,-2.8761834876,-1.0195250786,-1.592569035\C,-3.3173
029081,-0.2797719725,-0.4653274898\O,0.6067242819,-1.6231652494,-0.875
2261517\H,4.7519170978,0.0867673963,1.8862051474\H,3.7848826006,-2.163
207674,2.2928118045\H,1.8062699744,-2.9389639919,1.0055584108\H,-1.231
1665893,-1.9916744318,-2.5574640969\H,-3.5861137383,-1.2513254671,-2.3
781357884\H,-0.3338236321,-0.1983252068,1.2197889406\C,-2.8204210864,0
.7574313705,1.6859007983\C,-4.6543064575,0.1649017441,-0.3315020339\C,
-4.1189780397,1.173047612,1.7877594161\H,-4.4402821932,1.736574972,2.6
556020809\C,-5.0485534266,0.8748796473,0.7682259104\H,-2.1071441361,0.
9884054768,2.4690179049\H,-5.3621099567,-0.067729137,-1.1193119963\H,-
6.0738291132,1.2115591505,0.861852866\C,3.7415680488,3.8519632356,-1.1
453216417\C,3.1373275114,4.6304818543,-2.1328939223\C,4.838933943,4.36
11378154,-0.4500934831\C,3.6250681363,5.8964019599,-2.4179100074\H,2.2
872398417,4.2347917218,-2.670435701\C,5.3229621761,5.626335,-0.7380842
895\H,5.3033053009,3.75317559,0.3143741109\C,4.7174350337,6.3975646063
,-1.722706845\H,3.1496246629,6.4938086322,-3.1862458298\H,6.1755412959
,6.0129943744,-0.1931419898\H,5.0968797475,7.3872016392,-1.9472755183\
\Version=ES64L-G16RevC.01\State=1-A\HF=-1124.6037745\RMSD=7.847e-09\RM
SF=9.921e-07\Dipole=0.5073845,-0.3504308,1.1455149\Quadrupole=2.872112
3,1.3998336,-4.2719459,-0.1634195,6.1968038,-4.0974933\PG=C01 [X(C23H1
5N3O1)]\\@

2[4][1]

1\1\GINC-LOCALHOST\FOpt\RCAM-B3LYP TD-FC\6-311G(d,p)\C23H15N3O1\PIOTR\
02-Aug-2024\0\\#P CAM-B3LYP/6-311G(d,p) Fopt TD=(singlets,root=1, NSta
tes=3) SCF=tight #P Geom=(NoDistance,NoAngle) fcheck SCRF(Solvent=Ethy
lEthanoate)\\3H-benzotrazinyl naphth-2-oxy opt in S1 in AcOEt\\0,1\N,1
.6918903622,0.9679609113,-1.3234436758\N,3.8263094011,1.7911232656,0.1
106568092\C,3.2802881053,2.5621088639,-0.814666846\N,2.2202064329,2.08
2030418,-1.4836381951\C,2.1932192462,-1.9940139766,0.8107030243\C,1.63
42019503,-1.1689778618,-0.1512593655\C,2.1771461722,0.0901971441,-0.38
69397616\C,3.3099135715,0.553743893,0.3580473766\C,3.8467485893,-0.302
1048004,1.3191474942\C,3.2947030176,-1.5570768188,1.5349858435\C,-1.03
54890977,-0.3870267507,0.3805858161\C,-0.6776107155,-1.1406876684,-0.6
994401827\C,-1.6263087727,-1.5159337196,-1.6770254668\C,-2.3810679524,
0.0365671196,0.5288709209\C,-2.9233780475,-1.121488791,-1.5483452131\C
,-3.3429986933,-0.3332241217,-0.4457864928\O,0.5929117438,-1.617803324

,-0.9268443504\H,4.7044520443,0.0386973603,1.8831438518\H,3.7303592143
,-2.2067317781,2.2831950545\H,1.7613150919,-2.9727564401,0.9718251686\
H,-1.2920407929,-2.1143004241,-2.5147941345\H,-3.6535409216,-1.4072974
899,-2.296743367\H,-0.3071076505,-0.1065989187,1.1305049659\C,-2.79727
86163,0.8226896091,1.6315298103\C,-4.6835635132,0.0928094849,-0.290123
7738\C,-4.100010346,1.219063426,1.7555665111\H,-4.4041344218,1.8204840
768,2.6039594654\C,-5.0561896708,0.8513074212,0.7846168331\H,-2.063726
8667,1.1066170891,2.3776179263\H,-5.4119353079,-0.1936754678,-1.040601
1049\H,-6.0845592173,1.1730117034,0.8954762593\C,3.7803970707,3.903027
9926,-1.1316811102\C,3.1708324878,4.676108761,-2.1193660546\C,4.877428
9102,4.4093265611,-0.4372146396\C,3.6545819497,5.9409386891,-2.4069974
539\H,2.3184197781,4.2803560643,-2.6580722277\C,5.3579803704,5.6760598
987,-0.7289827238\H,5.3417286639,3.7998154828,0.3266728645\C,4.7489358
712,6.4439921689,-1.7127345867\H,3.1775315741,6.5372826446,-3.17490669
5\H,6.2108559197,6.0653557397,-0.1866340222\H,5.1258705755,7.433908092
6,-1.9394789219\\Version=ES64L-G16RevC.01\State=1-A\HF=-1124.5917791\R
MSD=7.554e-09\RMSF=2.430e-06\Dipole=0.2009094,0.4333047,0.397086\PG=C0
1 [X(C23H15N3O1)]\\@

2[4]^3

1\1\GINC-LOCALHOST\FOpt\UCAM-B3LYP\6-311G(d,p)\C23H15N3O1(3)\PIOTR\02-
Aug-2024\0\\#P UCAM-B3LYP/6-311G(d,p) FOpt=tight SCF=Direct freq(noram
an) #P Geom=(NoDistance,NoAngle) fcheck SCRF(Solvent=EthylEthanoate)\\
3Ph-benzotrazinyl naphth-2-oxy opt in ground state in AcOEt\\0,3\N,1.71
29404512,0.9949013421,-1.3513453789\N,3.8356438566,1.8161970729,0.1035
062744\C,3.3022930485,2.5729932624,-0.8225571309\N,2.2339207501,2.1183
506553,-1.5175559047\C,2.1655350514,-1.9572269607,0.7984621668\C,1.620
8460825,-1.1374786967,-0.171912557\C,2.1729312379,0.1206064075,-0.4082
724064\C,3.295664901,0.5745029525,0.3459474316\C,3.8214603047,-0.27500
65579,1.3169004038\C,3.2635308472,-1.5251481321,1.5359771353\C,-1.0499
454882,-0.3785603086,0.3680988275\C,-0.6921275604,-1.1299030556,-0.713
5392738\C,-1.6462821884,-1.5253357876,-1.6780301955\C,-2.4010927744,0.
0216783892,0.5316113712\C,-2.9483251469,-1.1530982333,-1.5348040252\C,
-3.3682478553,-0.368394445,-0.4297639275\O,0.5843603898,-1.5821927478,
-0.9580010011\H,4.6757650224,0.0664632157,1.8860260818\H,3.6862853248,
-2.1749298516,2.291202682\H,1.726748397,-2.9331822638,0.95995933\H,-1.
3119590105,-2.1211524074,-2.5176180417\H,-3.6825249177,-1.4546507776,-
2.2730162727\H,-0.3179657078,-0.0828099453,1.1084893246\C,-2.817396694
9,0.8044465974,1.6365477807\C,-4.7141268481,0.0344074728,-0.2589235933
\C,-4.1253147505,1.1782922527,1.7753871228\H,-4.4294607973,1.777427268
8,2.6253883213\C,-5.0868016554,0.7901132934,0.8177670126\H,-2.07977978
47,1.1040914191,2.3724169714\H,-5.4465181338,-0.2676179091,-0.99932328
74\H,-6.1192577099,1.0939796189,0.9403345034\C,3.818468475,3.914075166
2,-1.1359932104\C,3.2261753568,4.6869606708,-2.1329329997\C,4.90976898
49,4.4156754233,-0.4294551057\C,3.720937575,5.9487793012,-2.4190767094
\H,2.3782714272,4.2944659461,-2.6806000671\C,5.401618759,5.6780581477,
-0.7188552374\H,5.3601533116,3.8052845835,0.3417844403\C,4.8090224229,
6.4465949753,-1.7129416941\H,3.2570797256,6.5455051446,-3.1946903684\H
,6.2499971844,6.0642269871,-0.1673561898\H,5.1949956466,7.4334960436,-
1.937856973\\Version=ES64L-G16RevC.01\State=3-A\HF=-1124.5338327\S2=2.
028524\S2-1=0.\S2A=2.000397\RMSD=5.123e-09\RMSF=5.912e-07\Dipole=0.161
3165,0.4127475,0.3758058\Quadrupole=1.4553912,2.2706557,-3.7260469,3.9
513142,4.3583429,-3.9884498\PG=C01 [X(C23H15N3O1)]\\@

4[4]^3

1\1\GINC-LOCALHOST\FOpt\UCAM-B3LYP\6-311G(d,p)\C23H15N3O1(3)\PIOTR\02-
Aug-2024\0\\#P UCAM-B3LYP/6-311G(d,p) FOpt=tight SCF=Direct freq(noram
an) #P Geom=(NoDistance,NoAngle) fcheck SCRF(Solvent=EthylEthanoate)\\
3Ph-benzotrazinyl 2-naphthalene Ipso in T in AcOEt\\0,3\N,1.7921434131
,0.3326118532,0.6573530033\N,3.9403385836,-0.4789257606,2.1094425811\C
,2.8016119737,-0.0148466079,2.6308573298\N,1.6937336934,0.4032267254,1

.9890319347\C,3.7500099931,-0.5504554086,-2.12242079\C,2.7209348131,-0
.1087359357,-1.3273345427\C,2.8963310363,-0.1276587487,0.0361417997\C,
4.0041215853,-0.5480254336,0.7369893619\C,5.0631319733,-1.0005140213,-
0.0701989932\C,4.9149672948,-0.991095791,-1.451447836\C,-0.4436611405,
-0.1627021494,-0.2406577769\C,0.7576848544,0.7016405277,-0.3488368872\
C,0.465348525,2.1633046216,-0.3261006575\C,-1.7347970944,0.350734686,-
0.1539829671\C,-0.7798414597,2.6329351796,-0.2348415148\C,-1.938535977
6,1.7642278749,-0.1460107239\O,1.4739687579,0.3777283867,-1.6133649742
\H,5.9792144754,-1.3515875919,0.3865378785\H,5.7396945719,-1.342824581
3,-2.0590650167\H,3.6857336344,-0.5654396886,-3.2014668931\H,1.3228851
164,2.8212421447,-0.3926272862\H,-0.9515724937,3.7036267559,-0.2259373
537\H,-0.2720583938,-1.2323320355,-0.245217089\C,-2.8688005336,-0.4985
460048,-0.0685104203\C,-3.2289036607,2.2688739844,-0.0559111206\C,-4.1
351024717,0.0287221963,0.0198422978\C,-4.3221894792,1.4165215472,0.026
4723637\H,-3.3778462819,3.342579931,-0.0501655042\H,-5.3218383205,1.82
6654662,0.0964109982\C,2.7184802112,0.0592550135,4.1163107443\C,1.5756
788227,0.5485963365,4.7496081497\C,3.7949204994,-0.3649243248,4.893762
2439\C,1.5138408771,0.611448205,6.1328712922\H,0.7396951075,0.87802042
49,4.1483810302\C,3.730414549,-0.301121642,6.2772225911\H,4.6766975484
,-0.7430426543,4.3957168352\C,2.5902524965,0.1871418813,6.9015542185\H
,0.6210351413,0.9937404126,6.613097714\H,4.5739280289,-0.634106904,6.8
70089446\H,2.5402318635,0.2370776211,7.9827820408\H,-2.7184481533,-1.5
716790965,-0.0730321818\H,-4.99205816,-0.630187671,0.0850631448\\Versi
on=ES64L-G16RevC.01\State=3-A\HF=-1124.5364046\S2=2.090324\S2-1=0.\S2A
=2.004938\RMSD=7.203e-09\RMSF=7.261e-07\Dipole=-1.5506733,0.3665123,-0
.7760307\Quadrupole=7.5314524,-2.914295,-4.6171574,-4.0179738,-2.58202
14,1.1591839\PG=C01 [X(C23H15N3O1)]\\@

5[4]

1\1\GINC-LOCALHOST\FOpt\RCAM-B3LYP\6-311G(d,p)\C23H15N3O1\PIOTR\10-Aug
-2024\0\\#P CAM-B3LYP/6-311G(d,p) FOpt=tight SCF=Direct freq(noraman)
#P Geom=(NoDistance,NoAngle) fcheck SCRF(Solvent=EthylEthanoate)\\3Ph-
benzotrazinyl 2-naphthalene Ipso in cS in AcOEt\\0,1\N,0.1461824057,0.
6203852337,0.1009195265\N,2.7863590363,1.0976102759,-0.0872041018\C,2.
289338598,-0.1065366438,0.0154768624\N,0.9730352208,-0.3962910112,0.12
96083874\C,0.0910965787,4.2914092437,-0.2464424969\C,-0.5001885717,2.9
962710144,-0.142882531\C,0.5032876314,1.8958403918,-0.0415219491\C,1.9
321928681,2.1510109763,-0.1212981201\C,2.3847972752,3.4452387175,-0.23
52537962\C,1.4488466781,4.487190912,-0.2885587804\C,-1.8463102881,-0.4
84037852,-0.7308207335\C,-1.2378045489,0.1820614379,0.2833220204\C,-1.
8361979347,0.3670067715,1.5424374794\C,-3.1553348849,-0.9891397011,-0.
5394768163\C,-3.0894199016,-0.1257211275,1.7483933201\C,-3.7864281907,
-0.8081171839,0.718408214\O,-1.7134425857,2.7739668498,-0.1428620509\H
,3.4483304354,3.6261891459,-0.2904250881\H,1.8185039832,5.5031783941,-
0.3767333144\H,-0.5955685473,5.1257463332,-0.3067944109\H,-1.302600825
6,0.8928067484,2.3229883251\H,-3.5702853775,0.0028420437,2.7106552021\
H,-1.3454278746,-0.6219047325,-1.6805423117\C,-3.8473814686,-1.6753887
737,-1.5667071178\C,-5.0925282762,-1.3202929694,0.9087106381\C,-5.1081
078798,-2.1567820674,-1.352417171\C,-5.737431094,-1.9772434449,-0.1013
564492\H,-5.5732101198,-1.1810849717,1.8701583993\H,-6.7372513914,-2.3
639965803,0.0537705504\C,3.2036047665,-1.2731339055,0.0264576212\C,2.7
094919107,-2.5713720285,0.1506655881\C,4.5783238868,-1.0708998602,-0.0
909870868\C,3.5806551707,-3.6496466862,0.1566393823\H,1.6439411887,-2.
729653743,0.2429726947\C,5.4449454497,-2.1512712072,-0.0844440991\H,4.
9532103448,-0.0611051375,-0.1866479679\C,4.9486391488,-3.4430867674,0.
0392288958\H,3.1893208461,-4.654900129,0.2537182901\H,6.5113554954,-1.
9854768951,-0.1762758113\H,5.6275070207,-4.2873475041,0.0441613298\H,-
3.3614306932,-1.8108062187,-2.5259751925\H,-5.6315654856,-2.6790273482
,-2.1439023301\\Version=ES64L-G16RevC.01\State=1-A\HF=-1124.5845457\RM
SD=5.764e-09\RMSF=1.554e-06\Dipole=0.3341587,-1.4400423,0.1650431\Quad
rupole=6.2191259,-3.1663532,-3.0527727,7.6259853,-2.4080076,2.9652211\

PG=C01 [X(C23H15N3O1)]\\@

TS-1′

1\1\GINC-LOCALHOST\FTS\RCAM-B3LYP\6-311G(d,p)\C23H15N3O1\PIOTR\11-Aug-
2024\0\\#P CAM-B3LYP/6-311G(d,p) Opt(QST3, noeigentest) SCF=Direct Geo
m=(NoDistance,NoAngle) #P SCRF(Solvent=EthylEthanoate) fcheck freq(noR
aman)\\2-naphthylyoxy BT-Ph, zwitterion in AcOEt\\0,1\N,-0.16747634,0.
1549649675,0.0025780925\N,-2.5759237142,1.364740645,0.2760692734\C,-2.
4317729585,0.0706879145,0.1025579983\N,-1.2636595958,-0.5697134549,-0.
0628827545\C,0.7797833387,3.6986301281,-0.3774839263\C,1.0038684543,2.
3255504827,-0.2485660649\C,-0.1775194017,1.5329746629,0.0633204817\C,-
1.4691462587,2.1337159289,0.1744872405\C,-1.5926468555,3.5246062268,0.
1098838226\C,-0.4745545718,4.2736240338,-0.1813425556\C,2.1383356017,0
.1319205743,0.5128074227\C,1.0388365655,-0.5169891603,-0.0706443112\C,
1.1706133055,-1.7219910092,-0.8116719532\C,3.4281715832,-0.4993526157,
0.3869917466\C,2.3918805826,-2.2824075204,-0.964990935\C,3.5562011687,
-1.6852824208,-0.3686550191\O,2.1707955349,1.8156059736,-0.4304661357\
H,-2.572977034,3.9625857897,0.2309123521\H,-0.5651811438,5.3507796696,
-0.2638244718\H,1.6368900638,4.3134016643,-0.6179358273\H,0.2881445944
,-2.1502774686,-1.2653971014\H,2.511480334,-3.186416553,-1.5487607031\
H,1.9634953123,0.7290691875,1.4001348854\C,4.5554907842,0.0618284573,1
.006113607\C,4.8301188339,-2.2667224428,-0.4907564696\C,5.7922683796,-
0.5244487955,0.8710858826\C,5.9264331345,-1.6958804301,0.1141191959\H,
4.9383548861,-3.1770004813,-1.069153865\H,6.9008193434,-2.1577419566,0
.0084852552\C,-3.6403800631,-0.7904487518,0.0966322457\C,-3.5345395209
,-2.1785488248,0.0133378788\C,-4.9031396237,-0.2047069601,0.1760009307
\C,-4.6754511286,-2.9657307933,0.0076563807\H,-2.5559460482,-2.6349249
654,-0.0418684563\C,-6.0411424265,-0.994935107,0.1706223707\H,-4.97726
1747,0.8721161973,0.2407406139\C,-5.930697831,-2.3770361336,0.08595012
29\H,-4.5838660429,-4.0431719904,-0.0557725582\H,-7.0179508897,-0.5307
546058,0.2312987866\H,-6.821186435,-2.9940706215,0.0817350461\H,4.4387
017711,0.9730785523,1.5808344126\H,6.6597450585,-0.0844589933,1.346791
063\\Version=ES64L-G16RevC.01\State=1-A\HF=-1124.5591273\RMSD=7.290e-0
9\RMSF=3.399e-06\Dipole=-0.0691572,-0.649761,0.3186011\Quadrupole=7.69
4811,3.6466106,-11.3414216,-2.0409818,3.716463,4.6190599\PG=C01 [X(C23
H15N3O1)]\\@

6[4]

1\1\GINC-LOCALHOST\FOpt\RCAM-B3LYP\6-311G(d,p)\C23H15N3O1\PIOTR\03-Aug
-2024\0\\#P CAM-B3LYP/6-311G(d,p) FOpt=tight SCF=Direct freq(noraman)
#P Geom=(NoDistance,NoAngle) fcheck SCRF(Solvent=EthylEthanoate)\\3Ph-
benzotrazinyl naphtho-2-oxy Smiles product C1 in GS in EtOAc\\0,1\N,-2
.1019373795,-1.0272320197,0.3735453354\N,-4.7451525191,-0.2955101786,0
.7103618875\C,-4.2950830649,-1.533144988,0.7857161687\N,-3.0482702194,
-1.9753581244,0.6028389867\C,-1.969870916,2.4908459159,-0.7165684184\C
,-1.5655012421,1.2138396192,-0.3955995934\C,-2.4881273268,0.3088336244
,0.1345718207\C,-3.8366815452,0.6399549064,0.3129379943\C,-4.222114879
8,1.9568422209,0.0121481249\C,-3.304162484,2.8489112221,-0.4985352424\
C,0.1440413445,-0.2053995993,0.2777688899\C,-0.8257798097,-1.352747318
2,0.3385245977\C,-0.3651435835,-2.6966316372,0.4804956164\C,1.54666867
72,-0.6170113812,-0.0860074474\C,0.9434845838,-2.9770260439,0.34478000
87\C,1.9290647222,-1.9615303852,0.0062869333\O,-0.2917063132,0.8056649
706,-0.6402644393\H,-5.2578695816,2.2310656439,0.1614704865\H,-3.61949
27445,3.8570319019,-0.7387795057\H,-1.2514102588,3.1891225921,-1.12363
76899\H,-1.0963741724,-3.4665001203,0.6742025268\H,1.2871628891,-3.999
8351759,0.4429974449\H,0.1584435533,0.2617675621,1.2771419924\C,2.4749
452917,0.3445376337,-0.4560993633\C,3.2533668768,-2.3103326261,-0.2674
048603\C,3.7855198683,-0.0171895536,-0.735558001\C,4.1760697598,-1.346
7180713,-0.6359895298\H,3.5499352101,-3.3503918646,-0.1961239779\H,5.1
993557087,-1.6305994672,-0.8486724632\C,-5.2851952342,-2.6019144677,1.
1075828795\C,-4.9042877105,-3.9400764166,1.1994513735\C,-6.6199950493,

-2.2615939691,1.3200596754\C,-5.8417620227,-4.917503301,1.4965886444\H
,-3.86960249,-4.2062660423,1.0347814121\C,-7.5554347741,-3.239624285,1
.6201726242\H,-6.9063940876,-1.2218558777,1.2443616106\C,-7.1697606302
,-4.5708869495,1.7088956538\H,-5.5341029706,-5.9542303882,1.5638819562
\H,-8.5898371326,-2.9618944038,1.7839553949\H,-7.9012035411,-5.3355018
059,1.9422954537\H,2.1673887408,1.3795479263,-0.5312556917\H,4.5023604
468,0.7404151618,-1.027030269\\Version=ES64L-G16RevC.01\State=1-A\HF=-
1124.588753\RMSD=3.075e-09\RMSF=8.056e-07\Dipole=2.1227385,0.0333112,-
0.0057795\Quadrupole=6.1596925,7.5038224,-13.6635149,-0.7451912,-1.630
7989,-3.2139518\PG=C01 [X(C23H15N3O1)]\\@

1[4]-1H

1\1\GINC-LOCALHOST\FOpt\RCAM-B3LYP\6-311G(d,p)\C23H15N3O1\PIOTR\12-Aug
-2024\0\\#P CAM-B3LYP/6-311G(d,p) FOpt=tight SCF=Direct freq(noraman)
#P Geom=(NoDistance,NoAngle) fcheck SCRF(Solvent=EthylEthanoate)\\benz
otrazinyl 1,2-naphthyl-2H leuco C1\\0,1\N,0.0122782579,0.2393780573,-0
.3661924026\N,0.4359553953,2.9103368707,-0.2582405049\C,1.4021293586,2
.0986215711,-0.052145703\N,1.2249140705,0.7094352836,0.1929805033\C,-3
.4353587245,1.3916469997,0.2789448839\C,-2.364446742,0.5382131963,0.08
24369084\C,-1.0947132245,1.0556341561,-0.091751761\C,-0.8699794009,2.4
27196853,-0.0860662506\C,-1.9454179207,3.2890182975,0.1096507624\C,-3.
2169708561,2.7666610043,0.2933050283\C,-0.2025824098,-1.1500973471,-0.
3035768501\C,-1.4771348224,-1.6346626649,-0.1170108152\C,-1.7361361729
,-3.0254930753,-0.0927918639\C,-0.6491295375,-3.9243338803,-0.24858526
79\C,0.8696802144,-2.0563238129,-0.4591001723\C,2.7989010108,2.5746226
87,-0.0053494302\C,3.8661748228,1.6796797008,-0.0712633602\C,5.1723076
261,2.1452921387,-0.0168085698\C,5.422342869,3.5048856532,0.0988662801
\C,4.3614544727,4.4027966663,0.1618705516\C,3.0581668117,3.9420848793,
0.1135756099\O,-2.5803845376,-0.8179316181,0.0354113934\H,-1.766702098
,4.356354977,0.1132324434\H,1.8622492131,-1.6524469271,-0.6025235097\H
,3.6660163441,0.6223908003,-0.1791089031\H,5.9959571246,1.4440563499,-
0.0722548546\H,4.5545849866,5.4646095605,0.2542940653\H,2.2226486305,4
.6270179339,0.1675079782\H,-4.0558112915,3.4337259619,0.4453322424\H,-
4.425348423,0.9743705386,0.4102248948\H,6.4424401375,3.8675263396,0.13
93638248\C,-3.0430261698,-3.5425380239,0.0908517217\C,-3.2530208927,-4
.8925113902,0.1170631672\C,-0.9066479999,-5.3163650163,-0.2165049346\C
,-2.175128325,-5.7914624564,-0.0385866502\C,0.6535477016,-3.4025372043
,-0.4302670943\H,-4.2551829915,-5.2794319419,0.2571301639\H,-2.3593963
372,-6.8586357253,-0.0167148907\H,-3.8669080109,-2.8524206908,0.208672
0635\H,-0.0740280268,-6.0001299879,-0.3369470432\H,1.4835611332,-4.088
2389156,-0.5509188942\H,1.3030368042,0.497141842,1.1927615499\\Version
=ES64L-G16RevC.01\State=1-A\HF=-1124.6066676\RMSD=8.517e-09\RMSF=4.023
e-07\Dipole=0.8304745,-0.3691883,0.7907262\Quadrupole=10.3026806,3.589
7475,-13.8924281,1.1824807,-0.3756716,2.9241654\PG=C01 [X(C23H15N3O1)]
\\@

1[4]-H

1\1\GINC-LOCALHOST\FOpt\RCAM-B3LYP\6-311G(d,p)\C23H15N3O1\PIOTR\09-Aug
-2024\0\\#P CAM-B3LYP/6-311G(d,p) FOpt=tight SCF=Direct freq(noraman)
#P Geom=(NoDistance,NoAngle) fcheck SCRF(Solvent=EthylEthanoate)\\4-Ph
OxoNaphthalene helicene leuco form isomer\\0,1\N,-0.9833581259,-0.003
6024126,-0.6317978077\N,-3.2013608495,1.4371700193,-0.6122424587\C,-3.
2241647982,0.093402893,-0.264339574\N,-2.1699750888,-0.6280741816,-0.2
204890839\C,0.4451210861,3.3453734851,0.0921567906\C,0.359663581,1.975
994303,-0.1186813364\C,-0.862052184,1.3806120976,-0.3620615982\C,-2.01
64715622,2.1496294912,-0.3733638369\C,-1.953117167,3.5143539816,-0.145
2441193\C,-0.7127732567,4.1058472637,0.0743680263\C,0.1892386612,-0.76
16454699,-0.4443506083\C,1.3919313206,-0.1338420159,-0.2202992996\C,2.
59406707,-0.8660212336,-0.0846616434\C,0.1550048867,-2.1715116087,-0.5
288429798\C,2.5436354977,-2.281375272,-0.1734094155\C,1.2947509134,-2.
9082222962,-0.3950669804\O,1.5158193126,1.240238033,-0.1267041973\H,-2

.8587204314,4.1080522602,-0.1495761594\H,-0.6522548409,5.1745718707,0.
234925743\H,1.4169531603,3.7904484169,0.2595099918\C,-4.5298068116,-0.
5204888368,0.0643497036\C,-4.7465605215,-1.8696680117,-0.2163251157\C,
-5.5492152122,0.2220187519,0.658653999\C,-5.9596484888,-2.4620074948,0
.0907335858\H,-3.9553472722,-2.4416393021,-0.6821162\C,-6.7638455936,-
0.3747153465,0.9658959196\H,-5.3955345775,1.2635591701,0.9146422547\C,
-6.9729302884,-1.7160566718,0.6813334288\H,-6.1187631206,-3.5090063858
,-0.1367769253\H,-7.5450696711,0.2107409679,1.4348312784\H,-7.92231615
17,-2.18036181,0.9187446023\H,-4.0709121501,1.9442999353,-0.6052899054
\H,-0.8021135906,-2.6437999887,-0.694737134\H,1.2524815109,-3.98885854
33,-0.461967369\C,3.840649479,-0.2300794237,0.1426046232\C,3.745923455
7,-3.0161737665,-0.035268088\C,4.9806396344,-0.9724989403,0.2718953786
\C,4.9362211003,-2.3811447266,0.1814562133\H,5.8493538631,-2.954548622
3,0.2853727932\H,3.7030717574,-4.0973278403,-0.1047060215\H,3.87290823
73,0.8484884462,0.211245674\H,5.929014477,-0.4780352649,0.4449626216\\
Version=ES64L-G16RevC.01\State=1-A\HF=-1124.6203089\RMSD=9.447e-09\RMS
F=1.695e-06\Dipole=-1.7152025,0.6782379,0.2425731\Quadrupole=11.814002
4,6.3900131,-18.2040154,-4.4583389,-0.8902791,2.6356356\PG=C01 [X(C23H
15N3O1)]\\@

TS-3′

1\1\GINC-LOCALHOST\FTS\RCAM-B3LYP\6-311G(d,p)\C23H15N3O1\PIOTR\13-Aug-
2024\0\\#P CAM-B3LYP/6-311G(d,p) Opt(QST3, noeigentest, CalcFC) SCF=Di
rect Geom=(NoDistance,NoAngle) #P SCRF(Solvent=EthylEthanoate) fcheck
freq(noRaman)\\2-naphthylyoxy BT-Ph, zwitterion in AcOEt\\0,1\N,0.1152
629903,0.4936331252,0.1839942816\N,2.726435857,1.2159417782,0.06860914
11\C,2.3187585284,-0.0348485497,0.0878267436\N,1.0382442121,-0.4383724
868,0.1227198062\C,-0.2032973875,4.0905414946,-0.4771514604\C,-0.63392
47808,2.8076821601,-0.1695070532\C,0.401785822,1.8383981953,0.09008408
12\C,1.7818842331,2.1762419564,-0.0046996277\C,2.1448593602,3.50820592
89,-0.2469604509\C,1.1548784703,4.4276743515,-0.4960291879\C,-1.643262
4477,-1.1081900223,-0.2544120455\C,-1.2096074898,0.0979385202,0.279859
3366\C,-2.0983722494,1.0777155903,0.8588486721\C,-2.988409321,-1.43013
61056,-0.2348247773\C,-3.4700806304,0.6841235307,0.9184897442\C,-3.915
4051368,-0.49839233,0.3702536397\O,-1.8997344973,2.4963675673,-0.15480
34511\H,3.1954046461,3.7586809336,-0.2832234782\H,1.4263842703,5.45434
01963,-0.7121573529\H,-0.9600595716,4.8357009877,-0.6826670465\H,-1.70
15172572,1.6063063863,1.725337901\H,-4.1705068969,1.3417596947,1.41785
27112\H,-0.9223810727,-1.7702235886,-0.7154568377\C,-3.4835306974,-2.6
493523938,-0.7957321903\C,-5.3026815283,-0.8787059191,0.4060444725\C,-
4.8022964333,-2.9551081102,-0.7486957359\C,-5.7223006308,-2.0486997844
,-0.1306357291\H,-6.0092515689,-0.1986811866,0.8679032716\H,-6.7739305
007,-2.309286774,-0.0982273599\C,3.3312900324,-1.118233427,0.080222216
8\C,2.9548092293,-2.4563796726,0.1993201989\C,4.6819092072,-0.79831883
92,-0.0477800743\C,3.9153561039,-3.4558502298,0.1878997467\H,1.9084766
589,-2.7070894729,0.3055546473\C,5.6386438581,-1.8008387721,-0.0589230
818\H,4.9805015716,0.2368152189,-0.1393696791\C,5.2590503415,-3.131492
7435,0.0581171748\H,3.6127865954,-4.4915335344,0.2827053002\H,6.685460
3955,-1.5419408715,-0.160915915\H,6.0085742801,-3.9136590753,0.0492131
295\H,-2.7733621435,-3.3275311326,-1.2559776228\H,-5.1697404219,-3.881
6875941,-1.1715500594\\Version=ES64L-G16RevC.01\State=1-A\HF=-1124.540
202\RMSD=9.373e-09\RMSF=2.076e-04\Dipole=1.0639955,-0.2317269,0.098213
4\Quadrupole=1.7352114,8.2057287,-9.9409401,1.3271092,-2.8748293,2.415
9822\PG=C01 [X(C23H15N3O1)]\\@

7[4]

1\1\GINC-LOCALHOST\FOpt\RCAM-B3LYP\6-311G(d,p)\C23H15N3O1\PIOTR\03-Aug
-2024\0\\#P CAM-B3LYP/6-311G(d,p) FOpt=tight SCF=Direct freq(noraman)
#P Geom=(NoDistance,NoAngle) fcheck SCRF(Solvent=EthylEthanoate)\\3Ph-
benzotrazinyl naphtho-2-oxy Smiles product C3 in closed S in EtOAc\\0,
1\N,-1.692660926,0.8100264619,0.2170520865\N,-4.0127612515,2.294234941

1,0.3150892688\C,-2.7959871883,2.8066445619,0.3366634547\N,-1.62973084
97,2.1630021569,0.2594192724\C,-4.1902218127,-1.7958960432,-0.52383573
82\C,-2.9758039792,-1.1995675929,-0.2658057517\C,-2.9307194712,0.15865
29503,0.0718934797\C,-4.0886839312,0.9496624124,0.1158183596\C,-5.3193
283599,0.3117737353,-0.1120143105\C,-5.3554298869,-1.0269837882,-0.434
5664732\C,0.7106349154,0.6463898995,0.2488916344\C,-0.5810039036,0.085
7610602,0.2741020437\C,-0.7883238837,-1.3894320843,0.4716350834\C,1.81
70380619,-0.1571615334,0.230813852\C,0.4476237926,-2.1846045082,0.2598
763652\C,1.6691719294,-1.6197478703,0.1992111302\O,-1.8206738641,-1.90
42977689,-0.3873406972\H,-6.2198853449,0.9088512673,-0.0612820224\H,-6
.3100834665,-1.5037456873,-0.6210900075\H,-4.2192268291,-2.8469018895,
-0.7768465813\H,-1.1516253372,-1.5396884496,1.5056468676\H,0.321627116
4,-3.2593510436,0.2122508509\H,0.8045594206,1.7212902767,0.1938187445\
C,3.1470293103,0.392486105,0.1762410493\C,2.8763663288,-2.4206729411,0
.0832242386\C,4.2287740732,-0.4060652649,0.0853908277\C,4.0885936827,-
1.8441509313,0.0332366781\H,2.7675576531,-3.4982050171,0.0476568296\H,
4.980538662,-2.4533133121,-0.0469119475\H,3.2533767255,1.4704634971,0.
2057825099\H,5.2218598157,0.0240711848,0.0447927815\C,-2.6788951667,4.
2884997399,0.4547100016\C,-1.4354231286,4.9111692583,0.5566080563\C,-3
.8319134778,5.0715492299,0.4649067517\C,-1.3487466421,6.290690151,0.66
43586195\H,-0.5402461678,4.3051655027,0.5531877826\C,-3.7433575162,6.4
506887661,0.5735991828\H,-4.791795486,4.5803511888,0.3861639427\C,-2.5
017557706,7.064913286,0.6731228691\H,-0.3770530179,6.7634261019,0.7434
850748\H,-4.6469660046,7.0484705036,0.5795991189\H,-2.4326840835,8.142
9374073,0.7578416014\\Version=ES64L-G16RevC.01\State=1-A\HF=-1124.5549
122\RMSD=6.406e-09\RMSF=2.561e-07\Dipole=1.3619959,-1.2604788,0.232604
9\Quadrupole=8.3908812,5.990193,-14.3810742,1.236392,1.4190733,-0.5974
408\PG=C01 [X(C23H15N3O1)]\\@

phenoxazinyl

1\1\GINC-LOCALHOST\FOpt\UCAM-B3LYP\6-311G(d,p)\C12H8N1O1(2)\PIOTR\20-A
ug-2024\0\\#P UCAM-B3LYP/6-311G(d,p) FOpt=tight SCF=Direct freq(norama
n) #P Geom=(NoDistance,NoAngle) fcheck SCRF(Solvent=EthylEthanoate)\\P
henoxazinyl radical, Cs\\0,2\C,-0.0561147451,0.6644151271,-3.576304356
9\C,-0.0569999209,1.3726976816,-2.3966215406\C,-0.0561716351,0.7100895
399,-1.1523046341\C,-0.0544083251,-0.7006833212,-1.1690226811\C,-0.053
5119146,-1.4179656314,-2.3530427347\C,-0.0543661063,-0.7346304317,-3.5
571806312\C,-0.0544083251,-0.7006833212,1.1690226811\C,-0.0561716351,0
.7100895399,1.1523046341\C,-0.0569999209,1.3726976816,2.3966215406\H,-
0.0583529262,2.4551912792,2.3816663957\C,-0.0561147451,0.6644151271,3.
5763043569\C,-0.0543661063,-0.7346304317,3.5571806312\C,-0.0535119146,
-1.4179656314,2.3530427347\H,-0.0567717685,1.1900283658,-4.5228452385\
H,-0.0583529262,2.4551912792,-2.3816663957\H,-0.0521600038,-2.49958866
75,-2.3106405078\H,-0.0536726071,-1.2895539587,-4.4867148951\H,-0.0567
717685,1.1900283658,4.5228452385\H,-0.0536726071,-1.2895539587,4.48671
48951\H,-0.0521600038,-2.4995886675,2.3106405078\O,-0.0535310853,-1.40
24129135,0.\N,-0.0570731923,1.4314484677,0.\\Version=ES64L-G16RevC.01\
State=2-A'\HF=-591.8797079\S2=0.802643\S2-1=0.\S2A=0.752466\RMSD=5.230
e-09\RMSF=2.452e-06\Dipole=0.0008177,-0.6543452,0.\Quadrupole=-8.10530
77,-1.8944102,9.9997179,-0.007763,0.,0.\PG=CS [SG(N1O1),X(C12H8)]\\@

phenoxazine

1\1\GINC-LOCALHOST\FOpt\RCAM-B3LYP\6-311G(d,p)\C12H9N1O1\PIOTR\20-Aug-
2024\0\\#P CAM-B3LYP/6-311G(d,p) FOpt=tight SCF=Direct freq(noraman) #
P Geom=(NoDistance,NoAngle) fcheck SCRF(Solvent=EthylEthanoate)\\Pheno
xazine, Cs\\0,1\C,-0.207002143,0.6406151062,-3.5886763292\C,-0.0535281
251,1.3720119595,-2.4157747136\C,0.1225827018,0.7242940924,-1.20060786
12\C,0.1299278183,-0.6728136394,-1.1791149571\C,-0.0340864609,-1.40099
96361,-2.3381699108\C,-0.1968152757,-0.7437957794,-3.5545898177\C,0.12
99278183,-0.6728136394,1.1791149571\C,0.1225827018,0.7242940924,1.2006
078612\C,-0.0535281251,1.3720119595,2.4157747136\H,-0.0624094106,2.455

8827506,2.439413559\C,-0.207002143,0.6406151062,3.5886763292\C,-0.1968
152757,-0.7437957794,3.5545898177\C,-0.0340864609,-1.4009996361,2.3381
699108\H,-0.3358251919,1.1632860336,-4.5282481355\H,-0.0624094106,2.45
58827506,-2.439413559\H,-0.0233557528,-2.4818598792,-2.274489024\H,-0.
3166835438,-1.3181004991,-4.4641928695\H,-0.3358251919,1.1632860336,4.
5282481355\H,-0.3166835438,-1.3181004991,4.4641928695\H,-0.0233557528,
-2.4818598792,2.274489024\O,0.3330191315,-1.3612568416,0.\N,0.31079360
33,1.4079306639,0.\H,0.1239979025,2.3983386895,0.\\Version=ES64L-G16Re
vC.01\State=1-A'\HF=-592.5064784\RMSD=7.090e-09\RMSF=1.364e-06\Dipole=
-0.2956953,0.9627192,0.\Quadrupole=-8.5893383,5.2944559,3.2948823,-0.0
733695,0.,0.\PG=CS [SG(H1N1O1),X(C12H8)]\\@

6. References

(1) Bax, A.; Davis, D. G. MLEV-17-based two-dimensional homonuclear magnetization transfer spectroscop, *J. Magn. Res.* **1985**, *65*, 355–360.

(2) Hwang, T.-L.; Shaka, A. J. Cross relaxation without TOCSY: transverse rotating-frame Overhauser effect spectroscopy, *J. Am. Chem Soc.* **1992**, *114*, 3157–3159.

(3) PK, Gaussian 16, M. J. Frisch, G. W. Trucks, H. B. Schlegel, G. E. Scuseria, M. A. Robb, J. R. Cheeseman, G. Scalmani, V. Barone, G. A. Petersson, H. Nakatsuji, X. Li, M. Caricato, A. V. Marenich, J. Bloino, B. G. Janesko, R. Gomperts, B. Mennucci, H. P. Hratchian, J. V. Ortiz, A. F. Izmaylov, J. L. Sonnenberg, D. Williams-Young, F. Ding, F. Lipparini, F. Egidi, J. Goings, B. Peng, A. Petrone, T. Henderson, D. Ranasinghe, V. G. Zakrzewski, J. Gao, N. Rega, G. Zheng, W. Liang, M. Hada, M. Ehara, K. Toyota, R. Fukuda, J. Hasegawa, M. Ishida, T. Nakajima, Y. Honda, O. Kitao, H. Nakai, T. Vreven, K. Throssell, J. A. Montgomery, Jr., J. E. Peralta, F. Ogliaro, M. J. Bearpark, J. J. Heyd, E. N. Brothers, K. N. Kudin, V. N. Staroverov, T. A. Keith, R. Kobayashi, J. Normand, K. Raghavachari, A. P. Rendell, J. C. Burant, S. S. Iyengar, J. Tomasi, M. Cossi, J. M. Millam, M. Klene, C. Adamo, R. Cammi, J. W. Ochterski, R. L. Martin, K. Morokuma, O. Farkas, J. B. Foresman, and D. J. Fox, Gaussian, Inc., Wallingford CT. 2016,.

(4) Cossi, M.; Scalmani, G.; Rega, N.; Barone, V. New developments in the polarizable continuum model for quantum mechanical and classical calculations on molecules in solution, *J. Chem. Phys.* **2002**, *117*, 43–54.

(5) Fulmer, G. R.; Miller, A. J. M.; Sherden, N. H.; Gottlieb, H. E.; Nudelman, A.; Stoltz, B. M.; Bercaw, J. E.; Goldberg, K. I. NMR chemical shifts of trace impurities: Common laboratory solvents, organics, and gases in deuterated solvents relevant to the organometallic chemist, *Organometallics* **2010**, *29*, 2176–2179.

(6) Lucarini, M.; Pedrielli, P.; Pedulli, G. F.; Valgimigli, L.; Gigmes, D.; Tordo, P. Bond dissociation energies of the N-H bond and rate constants for the reaction with alkyl, alkoxyl, and peroxyl radicals of phenothiazines and related compounds, *J. Am. Chem Soc.* **1999**, *121*, 11546–11553.
